# SPECK: an unsupervised learning approach for cell surface receptor abundance estimation for single-cell RNA-sequencing data

**DOI:** 10.1093/bioadv/vbad073

**Published:** 2023-06-13

**Authors:** Azka Javaid, H Robert Frost

**Affiliations:** Department of Biomedical Data Science, Dartmouth College, Hanover, NH 03755, USA; Department of Biomedical Data Science, Dartmouth College, Hanover, NH 03755, USA

## Abstract

**Summary:**

The rapid development of single-cell transcriptomics has revolutionized the study of complex tissues. Single-cell RNA-sequencing (scRNA-seq) can profile tens-of-thousands of dissociated cells from a tissue sample, enabling researchers to identify cell types, phenotypes and interactions that control tissue structure and function. A key requirement of these applications is the accurate estimation of cell surface protein abundance. Although technologies to directly quantify surface proteins are available, these data are uncommon and limited to proteins with available antibodies. While supervised methods that are trained on Cellular Indexing of Transcriptomes and Epitopes by Sequencing data can provide the best performance, these training data are limited by available antibodies and may not exist for the tissue under investigation. In the absence of protein measurements, researchers must estimate receptor abundance from scRNA-seq data. Therefore, we developed a new unsupervised method for receptor abundance estimation using scRNA-seq data called SPECK (Surface Protein abundance Estimation using CKmeans-based clustered thresholding) and primarily evaluated its performance against unsupervised approaches for at least 25 human receptors and multiple tissue types. This analysis reveals that techniques based on a thresholded reduced rank reconstruction of scRNA-seq data are effective for receptor abundance estimation, with SPECK providing the best overall performance.

**Availability and implementation:**

SPECK is freely available at https://CRAN.R-project.org/package=SPECK.

**Supplementary information:**

Supplementary data are available at *Bioinformatics Advances* online.

## 1 Introduction

Transcriptome profiling has conventionally been performed using RNA sequencing (RNA-seq) of bulk tissue samples (Emrich *et al.*, 2007; Mortazavi *et al.*, 2008). While bulk RNA-seq facilitates genome-wide gene expression profiling, it measures the average gene expression for all cells within a tissue sample. To address the limitations of conventional RNA-seq, single-cell RNA-sequencing (scRNA-seq) technologies (Tang *et al.*, 2009), such as the 10× Chromium System (Zheng *et al.*, 2017), have been developed that can quantify gene expression within thousands of cells from a single tissue sample. The resolution provided by single-cell assays is critical for unraveling the biology of diseases associated with complex tissues, e.g. the tumor microenvironment, with important implications for advancing precision medicine, e.g. understanding the association between specific driver mutations and the composition and phenotype of tumor infiltrating immune cells. Key applications of scRNA-seq data include cataloging the cell types in a tissue (Aran *et al.*, 2019), reconstructing dynamic processes (Qiu *et al.*, 2017) and analyzing cell–cell signaling (Cabello-Aguilar *et al.*, 2020). Many of these tasks are based on the abundance of cell surface proteins, which follows the traditional use of immunohistochemical profiling for characterizing dissociated cells [e.g. Fluorescence Activated Cell Sorting (Bonner *et al.*, 1972)]. Although techniques, such as Cellular Indexing of Transcriptomes and Epitopes by Sequencing (CITE-seq) (Stoeckius *et al.*, 2017), can measure protein abundance for individual cells using barcoded antibodies, these types of data are uncommon, and target a limited number of proteins that have available antibodies.

If receptor protein measurements are not available for a given sample, researchers can estimate receptor abundance from scRNA-seq data using supervised or unsupervised approaches. Supervised methods, such as the single-cell Transcriptome to Protein prediction with deep neural network (cTP-net) tool (Zhou *et al.*, 2020), fit a predictive model on training data that captures both gene expression and protein abundance, e.g. joint scRNA-seq/CITE-seq, and utilize the trained model to generate estimates for target scRNA-seq datasets. While supervised methods can provide excellent predictive performance, they are only feasible if sufficient training data exist and may be susceptible to over-fitting and low transparency. For example, cTP-net only supports 24 immune-related receptors and uses a deep learning approach that makes model interpretation challenging. For cases where training data are not available, which includes many receptors that lack CITE-seq antibodies, an unsupervised approach that leverages RNA expression to determine protein levels must be employed. While the heterogeneity induced by processes such as transcriptional bursting (Liu *et al.*, 2016) may reduce the otherwise 56–84% reported protein variation accounted for by RNA expression measured using canonical quantification tools (Li *et al.*, 2014), advances in single-cell technology may make it increasingly more feasible to use RNA expression to quantify protein abundance. Abundance estimation using gene expression data is further justified in the context of relative abundance estimation. While post-transcriptional regulation has a substantial influence on absolute protein concentration, these processes do not have a major impact on relative protein levels (Jovanovic *et al.*, 2015).

One simple unsupervised strategy uses the expression of the associated RNA transcript as a proxy for protein abundance. While this approach can work in some situations, the sparsity of scRNA-seq data often leads to poor quality estimates. Although this sparsity can be mitigated by a cluster-based approach that approximates abundance using the average expression across all cells in a cluster, such a cluster-level analysis is sensitive to the number of computed clusters, and, importantly, ignores within-cluster heterogeneity. scRNA-seq data imputation techniques form a second promising family of unsupervised methods for generating cell-specific estimates. In this scenario, the imputed value of the receptor transcript is used to estimate protein abundance under the assumption that the imputation process reduces sparsity without inflating false positives. Although a large number of scRNA-seq imputation approaches exist (Hou *et al.*, 2020), we have found that the class of reduced rank reconstruction (RRR) techniques, which presumes that the intrinsic dimensionality of scRNA-seq data is much lower than the empirical rank, provides superior performance relative to other methods. RRR-based imputation methods include Markov Affinity-based Graph Imputation of Cells (MAGIC) (Dijk *et al.*, 2018) and Adaptively thresholded Low-Rank Approximation (ALRA) (Linderman *et al.*, 2022). While MAGIC and ALRA significantly outperform the naïve approach of directly using the receptor transcript, they have limitations. ALRA, e.g. applies the same quantile probability of 0.001 to threshold each gene, thereby overlooking considerable individual, gene-level expression variability. While MAGIC, like ALRA, is scalable, its performance is largely inferior to ALRA’s as a single-cell imputation strategy (Linderman *et al.*, 2022).

To address the limitations of existing unsupervised approaches for estimating relative receptor protein abundance, we developed a new technique named Surface Protein abundance Estimation using CKmeans-based clustered thresholding (SPECK). Similar to ALRA, the SPECK method utilizes a singular value decomposition (SVD)-based RRR but includes a novel approach for thresholding of the reconstructed gene expression matrix that improves receptor abundance estimation. A second important contribution of this article is a comprehensive evaluation of unsupervised receptor abundance estimation performance across at least 25 human cell surface receptors and multiple tissue types.

## 2 Methods

### 2.1 SPECK method for scRNA-seq data

**Figure 1.**
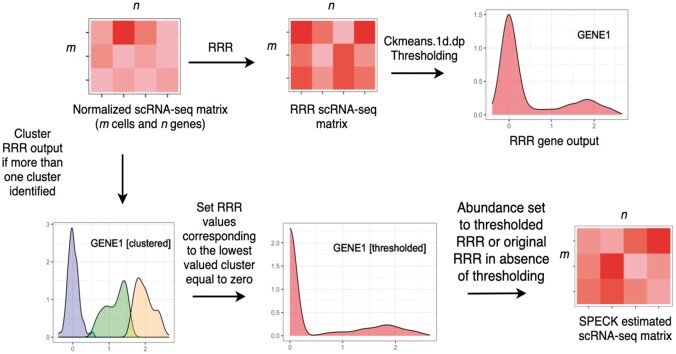
SPECK method: SPECK performs normalization, rank selection, RRR and thresholding on an *m *×* n* scRNA-seq count matrix with *m* cells and *n* genes. The reconstructed and thresholded matrix is of size *m *×* n*. To evaluate SPECK, receptor abundance estimates were visually assessed using feature plots and heat maps and correspondence to CITE-seq data was quantified using the Spearman rank correlation

The SPECK method estimates abundance profiles for *m* cells and *n* genes in a *m *×* n* matrix of scRNA-seq counts using the procedure outlined below. See Figure 1 and Algorithm 1 for the implementation overview and the associated pseudocode, respectively.

Algorithm 1 SPECK Algorithm

1: $X_{m,n}\overset{\text{Normalization}}{\to }X_{m,n}$

*Rank-k Selection and Singular Value Decomposition*

2: $\sum_{i\leftarrow 1}^{100}\mu_{i}\sigma_{i}v_{i}^{T}$       ▹ Rank-100 SVD of $X_{m,n}$.

3: $\text{rsdev}\leftarrow \sqrt{\frac{{\left(\sigma_{1},\sigma_{2},\ldots ,\sigma_{100}\right)}^{2}}{m-1}}$       ▹ PCs stdev.

4: rdiff←|rate of change(rsdev)|

5: rval←rle(rdiff).values     ▹ Run length encoding.

6: rlen←rle(rdiff).lengths

7: k←rdiff.index(min(rval≥0.01∧rlen≥2))+1 ▹ Rank-k.

8: $X_{m,n}\leftarrow \mu_{m,k}\times \sigma_{k,k}\times v_{k,n}^{T}$     ▹ Matrix reconstruction.

*Clustered Thresholding*

9: **for**v←1 to *n* **do**        ▹ Threshold each gene.

$\text{ckres}\leftarrow \mathrm{Ckmeans}.1d.dp(x=X_{m,n}[v],$ k=c(1:4))

10:   num←ckres.cluster

11:   val←ckres.centers

12:   **if**len(set(num))>1**then**

13:    ind←val.index(min(val))+1

14:    $X_{m,n}[v][\text{num}=\text{ind}]\leftarrow 0$

15:   **end if**

16: **end for**

### 2.2 Rank estimation and RRR

Given a $X_{m,n}$ scRNA-seq count matrix, whose elements represent the number of mRNA molecules associated with each gene detected in a specific cell, we first performed log-normalization using Seurat’s normalization pipeline (Satija *et al.*, 2015) to generate relative expression values. Seurat’s log-normalization procedure divides the gene-level counts for each cell by the total counts for all genes in that cell, multiplies this value by a scale factor of 10 000 adds a pseudocount of one and then natural-log transforms the product. A SVD-based RRR is next performed on the resulting $X_{m,n}$ normalized matrix to create a low-rank representation of the original gene expression data. Because genes act in a concerted and interdependent manner, the original expression values are well approximated by a low-rank matrix produced by the RRR procedure (Kapur *et al.*, 2016; Silver *et al.*, 2013).

To perform the RRR, we leveraged the randomized SVD algorithm implemented in the rsvd R package (Erichson *et al.*, 2019), which was observed to be accurate and computationally efficient relative to non-randomized truncated SVD techniques. This randomized SVD method was used to generate a rank-100 decomposition of the $X_{m,n}$ normalized expression matrix. To estimate the exact rank for the final RRR, the singular values from this rank-100 SVD decomposition were used to compute the standard deviation of the non-centered sample principal components and a rate of change in these standard deviations between successive components was subsequently computed. The target rank *k* was then selected to represent a numeric value, ranging from 1 to 100, for which the absolute value of the difference between consecutive standard deviation estimates was at least 0.01 for two or more estimate pairs. Although this rank selection procedure is a heuristic method, motivated by the commonly used elbow method for determining the number of principal components, we find that it works well in practice. The rsvd-based decomposition was subsequently used to generate a rank *k* reconstruction of the $X_{m,n}$ normalized expression matrix.

### 2.3 Cluster-based thresholding

We next performed clustered thresholding on the RRR values for each of the *n* genes from the $X_{m,n}$ RRR matrix. This step was inspired by the bimodality often exhibited by protein expression distributions, which functionally reflects the presence of two different sub-populations with varying individual response mechanisms to factors like stress or drug treatment (Blake *et al.*, 2006; Dobrzyński *et al.*, 2012). While bimodality in protein expression at the population level has historically been linked to stochastic switching (Acar *et al.*, 2008), defined by the transition of cells between multiple phenotypes as a result of environmental fluctuations, it is now more recently attributed to cell-to-cell variability that affects the sustained oscillation frequencies in a heterogeneous cell population (Dobrzyński *et al.*, 2012). We sought to leverage this idea of population-level bimodality exhibited by proteomics data in our abundance estimation technique by performing a thresholding step on the RRR values to ensure that the resulting estimates were representative of the corresponding CITE-seq data.

For this purpose, we utilized a 1D) clustering algorithm from the Ckmeans.1d.dp package (Song and Zhong, 2020; Wang and Song, 2011) to perform thresholding on the reconstructed expression values for each gene. This algorithm functions by reducing the overarching problem of clustering a 1D array consisting of $x_{1},\ldots ,x_{n}$ values into *k* clusters to a sub-problem of minimizing the sum of squares of within-cluster distances from an element to its associated cluster mean for clustering $x_{1},\ldots ,x_{i}$ values into *m* clusters. Dynamic programming is then used to solve this recurrence equation and find the assignment of all *n* values to *k* clusters. As noted, this iterative computation reduces the time complexity of the algorithm to *O*(*nk*), which compares with *O*(*qknp*) for standard *k*-means algorithm, where *q* defines the number of iterations and *p* specifies the dimensionality (Manning *et al.*, 2008; Wang and Song, 2011). We selected this dynamic programming-based 1D clustering algorithm since in addition to its runtime efficiency, it is optimal, and, unlike comparative methods like *k*-means, is not dependent on the definition of initial cluster centers, thereby producing consistent cluster assignments for each run. The Ckmeans.1d.dp algorithm implementation requires specification of *k*, which defines the minimum and maximum number of clusters to be examined. While given the bimodal expression pattern of proteomics data, an upper bound of two seems reasonable for this Ckmeans.1d.dp-based parameter *k*, we specified a slightly higher upper bound to account for potential additional expression multimodality in CITE-seq data beyond that accounted for by bimodality. We found that the upper limit of four was appropriate to ensure that cells were mapped to a fairly granular and interpretable number of clusters. Further analysis revealed that the thresholding performance was relatively insensitive to this upper bound, as specified within the range of 3–15. With the minimum cluster number set to one and the maximum cluster number set to four, we performed thresholding only if more than one cluster was identified. If so, then all the non-zero values in the RRR output for the gene corresponding to the indices of the least-valued cluster, as identified by the cluster mean, were set to zero. The zero values corresponding to the indices of the least-valued cluster and all other non-zero and zero values corresponding to higher-valued clusters in the RRR gene output were preserved. If only one cluster was identified, thresholding was not performed and the RRR gene values were preserved. The final output consisted of a $X_{m,n}$ RRR and thresholded matrix.

## 3 Evaluation

### 3.1 Comparison methods

Comparative evaluation of the SPECK method was performed against four other approaches described below, which include three unsupervised approaches and one supervised receptor abundance estimation strategy.

- ALRA: Receptor abundance was estimated using the imputed value of the associated transcript generated by the ALRA method with v.1.0.0 (Linderman *et al.*, 2022). scRNA-seq data were normalized using ALRA’s procedure, which normalizes each cell column by a factor of 10 000 followed by adding a pseudocount of one and taking the logarithm of each entry. Log-normalized data were then RRR and imputed using ALRA’s rank estimation approach, which chooses the largest value of *k*, given an upper bound of *k*<100, such that the gap between consecutive singular values *s_k_* is significantly different from the mean and standard deviation of typical noise spacings, defined to be $s_{80}\ldots s_{100}$ (i.e. $s_{k}>\mu +6\sigma$).
- MAGIC: Receptor abundance was estimated using the imputed value of the associated transcript generated by the MAGIC method with Rmagic v.2.0.3 (Dijk *et al.*, 2018). scRNA-seq data were normalized using the library size (i.e. transcript abundances) to ensure that each cell has the same transcript count. Normalized data were subsequently imputed with the number of nearest neighbors parameter *knn* set to 15.
- RNA transcript: Receptor abundance was estimated using the normalized value of the associated transcript with the normalization performed using Seurat’s log-normalization procedure (Satija *et al.*, 2015). This process entails dividing each cell by the total cell molecule count. A product of this value with a scaling factor is then computed followed by a pseudocount addition of one and a natural logarithm computation.
- cTP-net: Receptor abundance was estimated using the imputed value of the associated transcript generated by the cTP-net method with cTP-net v.1.0.3 (Zhou *et al.*, 2020).

### 3.2 Public single-cell data

SPECK was evaluated relative to the ALRA, MAGIC and RNA transcript methods on four publicly accessible joint-CITE-seq/scRNA-seq datasets listed in Table 1: (i) Hao *et al.* (2021) human peripheral blood mononuclear cell (PBMC) dataset [GEO (Edgar *et al.*, 2002)] series Chromium 3’ with 211 TotalSeq A antibodies, (ii) the Stuart *et al.* (Stoeckius *et al.*, 2017) human bone marrow mononuclear cell (BMMC) dataset (GSE128639) that contains 33 454 cells similarly profiled with 25 TotalSeq A antibodies, (iii) the Lakkis *et al.* (2022) human blood monocyte and dendritic cell dataset profiled with 238 antibodies and (iv) the Gayoso *et al.* (2021)*Mus musculus* dataset (GSE150599) that contains 32 648 cells profiled with 102 mouse antibodies. Each dataset was individually processed using Seurat v.4.1.0 (Butler *et al.*, 2018; Hao *et al.*, 2021; Satija *et al.*, 2015; Stuart *et al.*, 2019) in R v.4.1.2 (R Core Team, 2021). CITE-seq ADT counts were normalized using the Seurat implementation of the centered log-ratio transformation (Aitchison, 1989).

**Table 1.** Joint scRNA-seq/CITE-seq datasets used for method evaluation

Organism	Source tissue	Number of analyzed cells	Number of antibodies	Reference
Human	PBMC	60 000	211	Hao *et al.* (2021)
Human	BMMC	30 000	25	Stoeckius *et al.* (2017)
Human	Monocytes	37 000	238	Lakkis *et al.* (2022)
Mouse	Spleen and Lymph Nodes	20 000	102	Gayoso *et al.* (2021)

### 3.3 Setup and evaluation metrics

We evaluated the SPECK-estimated abundance profiles on four different joint scRNA-seq/CITE-seq datasets detailed above and listed in Table 1.

We determined the upper limit of 60 000 analyzed cells by the ability to perform RRR on 16 CPU cores without any virtual memory allocation problems. From the initial antibodies included in each data, antibodies mapping to multiple HUGO Gene Nomenclature Committee (Tweedie *et al.*, 2021) symbols were removed. We also explored performance for PBMC and BMMC datasets for different random cell subsets with subset size varying from 1000 up to the maximum number of analyzed cells. For the subset analyses, which are included in the Supplementary Material, receptors not expressed in smaller cell subsets were dropped.

For each dataset, we measured the correspondence between the estimated receptor abundances and the CITE-seq ADT measurements using a variety of correlation and error metrics. For the results in the main manuscript, Spearman correlation was chosen since it can quantify relationships between the ranks of two variables, as opposed to their absolute values, which is appropriate for comparing the estimated relative abundance profiles produced by the SPECK, ALRA, MAGIC and the RNA transcript methods against CITE-seq data. The Spearman correlation metric is also robust to outliers and can be used to analyze non-linear relationships (Schober *et al.*, 2018). In addition to the Spearman rank correlation, we primarily assessed SPECK’s performance relative to the ALRA, MAGIC and the RNA transcript approaches using the Kendall’s rank correlation coefficient and the Pearson correlation metric, which can be leveraged to identify potential linearity in the relative abundance estimates (Freedman *et al.*, 2007).

## 4 Results

### 4.1 SPECK improves correspondence of abundance estimates with CITE-seq ADT data

**Figure 2.**
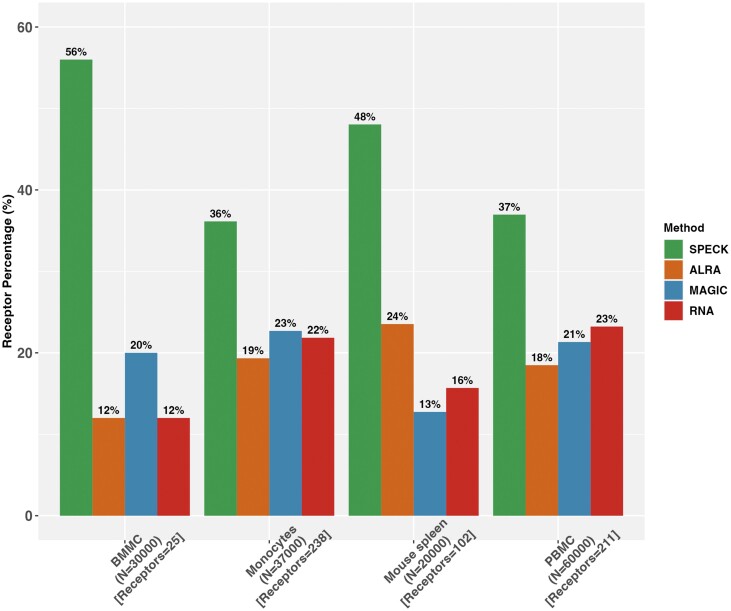
Proportion of receptors with the highest Spearman rank correlation values between CITE-seq ADT data and abundance estimates generated by the SPECK, ALRA, MAGIC or RNA transcript methods with number of cells specified to be 30 000 for the BMMC data, 37 000 for the Monocytes data, 20 000 for the Mouse Spleen and Lymph Nodes data and 60 000 for the PBMC data

**Figure 3.**
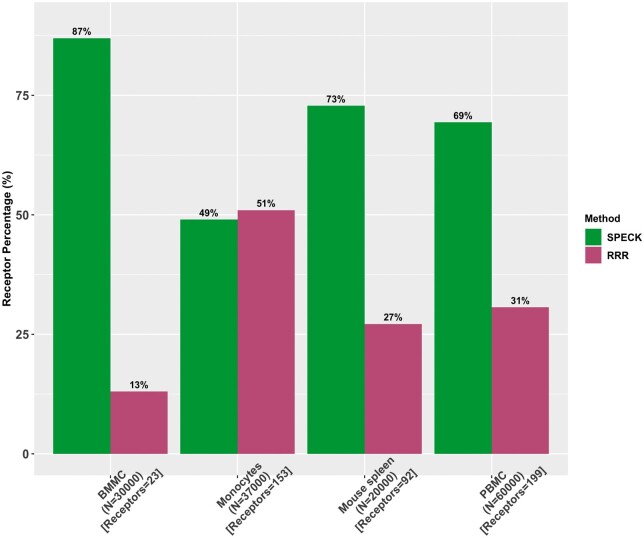
SPECK-based RRR and thresholded estimates versus SPECK-based RRR-only values and proportion of receptors with the highest rank correlation between CITE-seq ADT data and the estimated values for a subset of 30 000 cells for the BMMC data, 37 000 cells for the Monocytes data, 20 000 cells for the Mouse Spleen and Lymph Nodes data and 60 000 cells for the PBMC data

**Figure 4.**
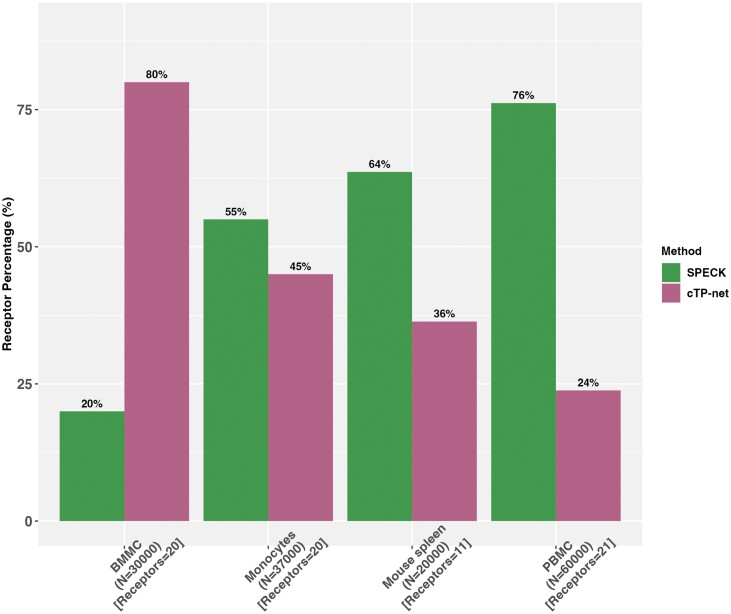
SPECK-based RRR and thresholded estimates versus cTP-net imputed values and proportion of receptors with the highest rank correlation between CITE-seq ADT data and the estimated values for a subset of 30 000 cells for the BMMC data, 37 000 cells for the Monocytes data, 20 000 cells for the Mouse Spleen and Lymph Nodes data and 60 000 cells for the PBMC data

For each evaluated method and dataset, we computed the proportion of evaluated receptors for which the abundance estimate generated by the method under investigation has the highest correlation with the corresponding CITE-seq data as compared to alternative estimation techniques. These results for the Spearman rank correlation are displayed in Figures 2 and 3. As observed in Figure 2, SPECK generates the best estimate for a larger proportion of receptors than ALRA, MAGIC or the normalized RNA transcript approach across all four datasets. Similarly, as visualized in Figure 3, when SPECK is directly compared to the RRR approach (i.e. SPECK without thresholding), SPECK has superior performance for three of the datasets with very similar performance on the monocytes data. We additionally compared SPECK to the cTP-net algorithm to perform assessment against a supervised receptor abundance estimation approach. As indicated by Figure 4, we note that there is a greater percentage of receptors that are highly correlated with the corresponding CITE-seq data, when estimated using the SPECK method versus when imputed using the cTP-net approach, based on the Spearman rank correlation metric for all datasets, except the BMMC data.

Supplementary Figures S1–S4 display analogous results using Spearman rank correlation for different cell subset size ranging from 5000 to 60 000 for the PBMC data and from 1000 to 30 000 for the BMMC data. These subset results demonstrate that SPECK’s relative performance benefit is relatively insensitive to dataset size. The superior performance of SPECK relative to ALRA, MAGIC and the RNA transcript approach is also found when using the Pearson correlation metric (Supplementary Figs S5 and S6) and the Kendall’s Tau correlation coefficient (Supplementary Figs S7 and S8). Lastly, while we additionally quantify the proportion estimate using the mean squared error (MSE) and the mean absolute error (MAE) in the supplement, we note that the MSE and the MAE are less appropriate metrics to gauge SPECK’s performance due to two primary reasons. First, the MSE and MAE are traditionally used to quantify absolute correspondence between the estimated abundance profiles and the corresponding CITE-seq data and second, the MSE and MAE are more appropriate metrics if we were leveraging pre-trained associations. Since SPECK is primarily an unsupervised receptor abundance estimation approach that does not leverage pre-trained weights and, moreover, quantifies differences on the relative scale, we highly recommend its evaluation using measures of correlation, especially using the Spearman rank correlation.

### 4.2 Examining individual abundance values using density estimation

We visually assessed correspondence between the estimated abundance profiles produced by SPECK, ALRA, MAGIC and the RNA transcript method with analogous CITE-seq ADT data using kernel density estimation-based smoothed scatter plots for random subsets of 10 000 cells from the PBMC and the BMMC datasets. This subset size was selected since 10 000 cells present a probable cutoff point between a small-sized and a comparatively large scRNA-seq dataset. The CD14, CD19 and CD79b receptors were chosen for visualization since each receptor functions as an important marker for an immune cell population. While CD14 is a marker for monocytes, types of leukocytes that contribute to an immune response by differentiating as macrophages and dendritic cells, CD19 and CD79b are markers for B cells, types of lymphocytes that are primarily involved in producing antibodies against foreign antigens and serving as antigen-presenting cells.

**Figure 5.**
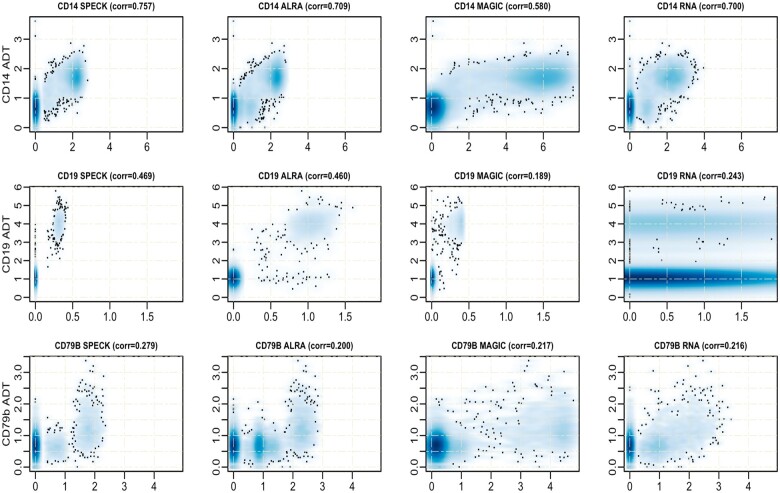
Smoothed scatter plot representation of correspondence between CITE-seq ADT measurements for CD14, CD19 and CD79b and receptor abundance profiles generated by SPECK, ALRA, MAGIC and the RNA transcript method for the PBMC data

**Figure 6.**
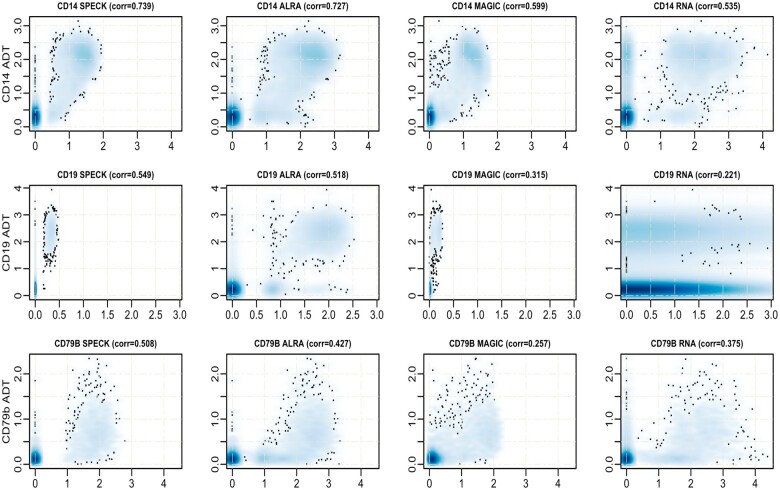
Smoothed scatter plot representation of the correspondence between CITE-seq ADT measurements for CD14, CD19 and CD79b and receptor abundance profiles generated by SPECK, ALRA, MAGIC and the RNA transcript method for the BMMC data

Figures 5 and 6 display these scatter plots for select receptors and, overall, indicate that compared to the abundance profiles generated by ALRA, MAGIC and the RNA transcript method, SPECK-estimated abundance profiles for CD14, CD19 and CD79b show a distinctive separation of cells in high-density regions that are highly correlated with CITE-seq data. The analogous plots for the Monocytes and the Mouse Spleen and Lymph Nodes datasets are indicated by Supplementary Figures S20 and S22, respectively. These results underscore SPECK’s important contribution in generating relatively accurate receptor abundance estimates as compared to other unsupervised techniques.

### 4.3 Visualization of individual estimates using a low-dimensional space

**Figure 7.**
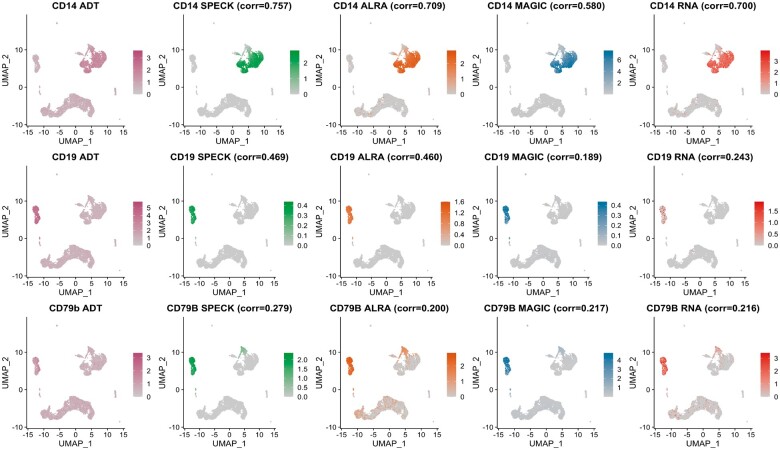
Low-dimensional projection of abundance profiles for CD14, CD19 and CD79b receptors as estimated by SPECK, ALRA, MAGIC and the RNA transcript method and corresponding CITE-seq ADT data for a subset of 10 000 cells from the PBMC data

**Figure 8.**
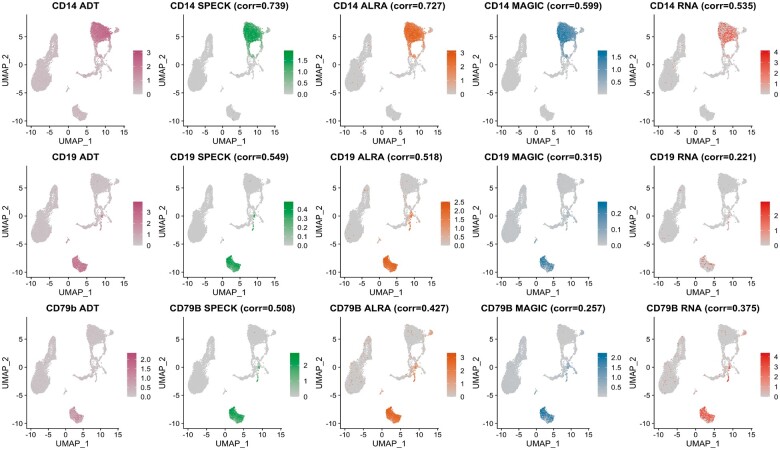
Low-dimensional projection of abundance profiles for CD14, CD19 and CD79b receptors as estimated by SPECK, ALRA, MAGIC and the RNA transcript method and corresponding CITE-seq ADT data for a subset of 10 000 cells from the BMMC data

We visually assessed correspondence between the estimated abundance profiles and CITE-seq ADT data via projection of cells onto the first two Uniform Manifold Approximation and Projection (UMAP) (Satija *et al.*, 2015) dimensions. This visualization allowed us to capture the estimated abundance profiles on an underlying low-dimensional manifold, thereby enabling mapping of these values over both the local and global topological structure of the transcriptomic data. Figures 7 and 8 display feature plot visualizations of ADT data and the estimated abundance profiles produced by SPECK, ALRA, MAGIC and the RNA transcript techniques for the CD14, CD19 and CD79b receptors from the PBMC and the BMMC datasets, respectively. The abundance estimates produced by SPECK have more visually defined expression profiles that are primarily relegated to region of cells with high CITE-seq ADT abundance. This trend is especially visible for the CD79b receptor for both the PBMC and the BMMC datasets, for which ALRA and the RNA transcript techniques especially indicate a homogeneous and less distinctive expression pattern. The analogous UMAP plots for the Monocytes and the Mouse Spleen and Lymph Nodes datasets are displayed by Supplementary Figures S19 and S21, respectively. These results suggest potential utility of the SPECK method for cell typing of scRNA-seq data.

### 4.4 Framework for selection of an informed abundance estimation strategy

**Figure 9.**
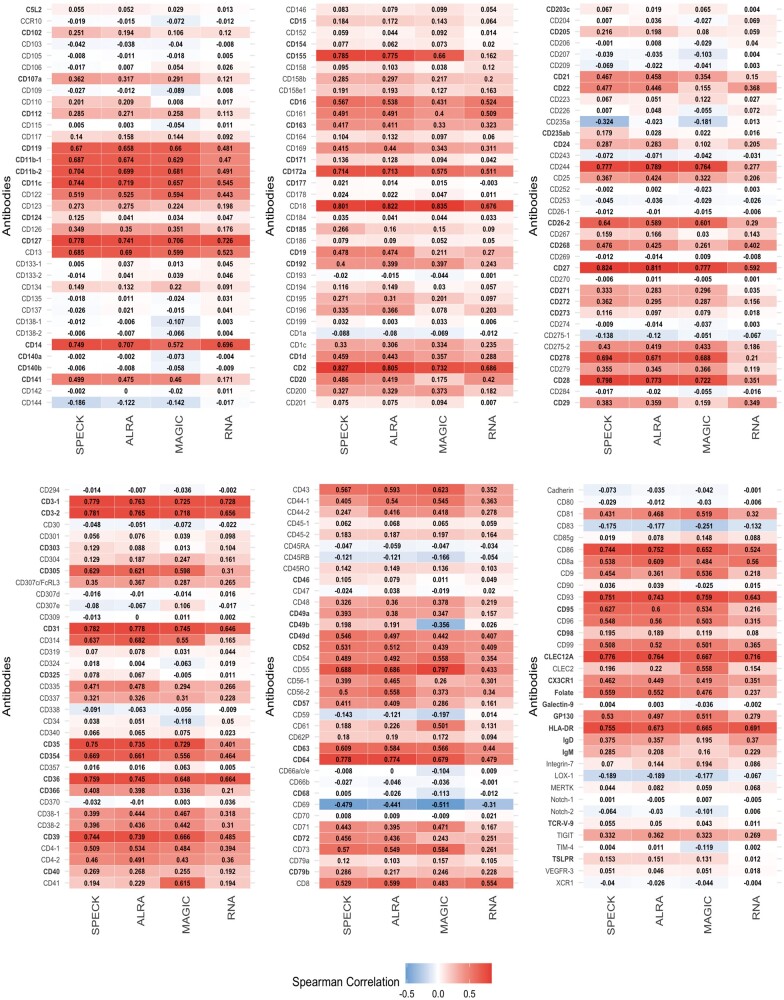
Individual rank correlations between CITE-seq ADT data and estimates generated by SPECK, ALRA, MAGIC and the RNA transcript method, averaged over a random subset of 60 000 cells for 215 receptors from the PBMC data. Bold text format is used to indicate receptors where the SPECK estimate has the largest correlation with CITE-seq ADT data as compared to the estimates generated by ALRA, MAGIC and the RNA transcript methods. Limits of the gradient color scale are determined by the minimum and maximum average correlation values for all methods combined

**Figure 10.**
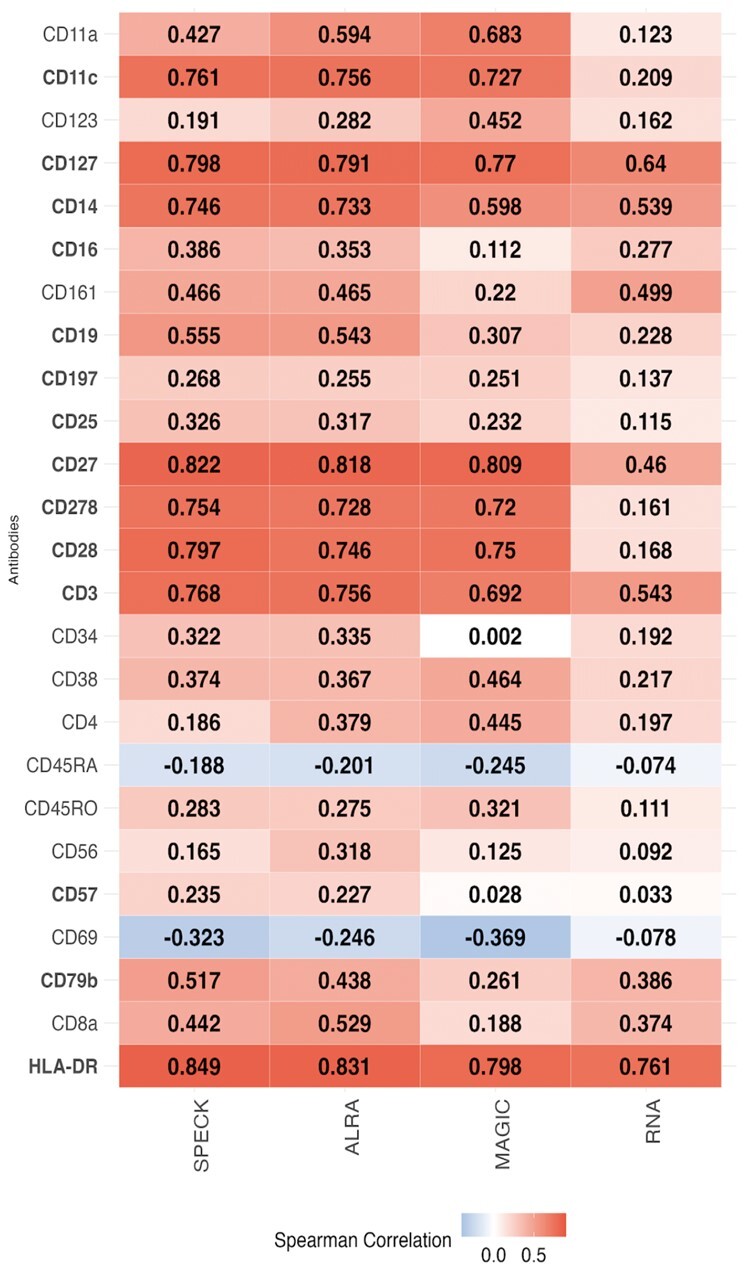
Individual rank correlations between CITE-seq ADT data and estimates generated by SPECK, ALRA, MAGIC and the RNA transcript method, averaged over a random subset of 30 000 cells for 25 receptors from the BMMC data

Following the overall evaluation of abundance profiles estimated by SPECK across all subsets and an examination of the distribution of estimated abundance values for select receptors for an individual cell subset, we quantified the magnitude and direction of the rank correlation values between CITE-seq ADT data and the estimated profiles generated by SPECK, ALRA, MAGIC and the RNA transcript method. Figure 9 shows the heatmap plot of these correlation values for 215 receptors averaged over a subset of 60 000 cells from the PBMC data while Figure 10 shows the heatmap for 25 receptors averaged over a subset of 30 000 cells from the BMMC data. In addition to confirming the results from Figure 2, which indicate that SPECK overall outperforms ALRA, MAGIC and the RNA transcript method in the task of abundance estimation, these figures have scientific utility as they can be referenced to determine the most appropriate estimation/imputation strategy for a given receptor. For example, for the CD8a receptor, estimated abundance profiles produced by ALRA are more highly correlated with corresponding ADT data compared to estimated abundance profiles produced by SPECK, MAGIC and the RNA approach for both the PBMC and the BMMC datasets. ALRA, may, therefore, be a preferable strategy for estimating abundance profiles for select receptors, such as CD8a as compared to alternative techniques. The analogous Pearson correlation-based results for the SPECK versus ALRA/MAGIC/RNA comparison for the PBMC data and the BMMC datasets are displayed by Supplementary Figures S13 and S14. Lastly, corresponding heatmaps for the Monocytes dataset are displayed by Supplementary Figures S15 and S16 and by Supplementary Figures S17 and S18 for the Mouse Spleen and Lymph Nodes dataset for the Spearman and Pearson correlation coefficients, respectively.

## 5 Discussion

In this work, we describe a new unsupervised learning method, SPECK, that uses RRR and thresholding to estimate cell surface receptor abundance from scRNA-seq data. We evaluate our approach on three joint scRNA-seq/CITE-seq datasets (PBMC, BMMC and Monocytes) for a large set of human receptors. In addition, we evaluated SPECK using the Mouse Spleen and Lymph Nodes dataset for ∼100 receptors, which further indicates SPECK’s potential as a receptor abundance estimation strategy for both human and the mouse specimens. This comparative evaluation demonstrates that SPECK generates more accurate receptor abundance estimates over the RRR-based imputation methods, including ALRA and MAGIC, or the direct use of normalized RNA transcript.

An important contribution of SPECK is a novel strategy for cluster-based thresholding of the reconstructed gene expression values. Our proposed thresholding mechanism differs from existing thresholding schemes, e.g. the ALRA thresholding approach, in two ways. First, our strategy does not always threshold a gene, and, second, it does not necessarily apply the same quantile to threshold each gene, thereby enabling recovery of more accurate, estimated abundance profiles. A second important contribution of this work is an extensive evaluation strategy for benchmarking the effectiveness of unsupervised methods for estimating receptor abundance using scRNA-seq data. With comparisons performed across multiple datasets from different tissue sources and species, this evaluation strategy provides considerable information on the relative performance of unsupervised methods across a wide range of receptors.

One limitation of our proposed approach is that SPECK is not necessarily the best abundance estimation strategy for all receptors. For example, SPECK produces a negative correlation value for CD69 for both the PBMC and the BMMC datasets as indicated by Figures 9 and 10, respectively. While this correlation is in agreement with the negative correlations returned by comparative methods, it still points to a deficiency in the use of transcriptomics data to infer protein abundance. We hope to address this limitation in future work by using pathway analysis to identify the biological characteristics of receptors whose abundance can be accurately estimated from scRNA-seq using unsupervised methods, such as SPECK. Such insights could then be leveraged by researchers to help determine whether unsupervised estimation of receptor abundance is feasible for a given investigation or whether direct proteomic measurements are motivated. A second limitation of our technique is that the thresholding step applied following the RRR approach is not always optimal. For example, as Figure 3 indicates, for the 37 000-cell subset of the Monocytes dataset, the aggregate percentage of receptors that are most correlated with analogous CITE-seq data when estimated using the complete SPECK method (i.e. RRR followed by thresholding) is ∼49%. In comparison, the aggregate percentage of receptors that are most correlated with CITE-seq data when estimated using the SPECK method without thresholding (i.e. RRR) is ∼51%. A third limitation of our approach stems from a key deficiency of scRNA-seq data, namely, that current scRNA-seq protocols can only capture static transcriptional states of cells at a specific time point. This limitation prevents SPECK’s applicability to surface protein abundance estimation for dynamic biological processes, such as tissue regeneration. The current R package implementation for SPECK is available on the Comprehensive R Archive Network (CRAN) (Javaid and Frost, 2022).

## 6 Conclusion

In conclusion, SPECK is a promising approach for unsupervised estimation of surface receptor abundance for scRNA-seq data, which addresses limitations of existing imputation methods, such as ALRA and MAGIC. The cell surface receptor abundance profiles generated by SPECK have important scientific utility for the analysis of single-cell data with specific relevance to the identification of the cell (sub)types, cell phenotypes and cell–cell signaling present in a tissue. Improved support for these single-cell analysis tasks will have a meaningful impact on basic research supporting precision medicine applications, especially in the immunology domain.

## Supplementary Material

vbad073_Supplementary_Data

## Data Availability

An implementation of the SPECK method and associated vignettes are available via the SPECK R package on CRAN (https://cran.r-project.org/web/packages/SPECK/index.html). The human PBMC CITE-seq data used to generate the results is publicly accessible from GSE164378. The human BMMC CITE-seq data used to generate the results is publicly accessible from GSE128639. The human blood monocyte and dendritic cell data used to generate the results is publicly accessible from https://upenn.app.box.com/s/64c9fsex50g1bhv67893cpdg9c5jqjzo. The mouse spleen and lymph nodes data used to generate the results is publicly accessible from GSE150599.
