# Supplementary material for: SPECK: an unsupervised learning approach for cell surface receptor abundance estimation for single-cell RNA-sequencing data
Source: Bioinform Adv. 2023 Jun 13;3(1):vbad073. doi: 10.1093/bioadv/vbad073 (PMC10290233; doi:10.1093/bioadv/vbad073)
Supplement: vbad073_Supplementary_Data [file vbad073_supplementary_data.pdf]

Supplementary Text

Azka Javaid and H. Robert Frost

May 23, 2023

Contents

1 Supplemental Methods **2**

List of Figures

S1 Proportion of receptors with the highest rank correlation values between CITE-seq ADT data and abundance estimates produced by either the SPECK, ALRA, MAGIC or RNA transcript methods with the number of cells ranging from 5,000 to 60,000 for the PBMC data. 3
S2 Proportion of receptors with the highest rank correlation values between CITE-seq ADT data and abundance estimates produced by either the SPECK, ALRA, MAGIC or RNA transcript methods with the number of cells ranging from 1,000 to 30,000 for the BMMC data. 3
S3 SPECK-based RRR and thresholded estimates versus SPECK-based RRR-only values and proportion of receptors with the highest Spearman rank correlation between CITE-seq ADT data and the estimated values for the PBMC data. 4
S4 SPECK-based RRR and thresholded estimates versus SPECK-based RRR-only values and proportion of receptors with the highest Spearman rank correlation between CITE-seq ADT data and the estimated values for the BMMC data. 4
S5 Proportion of receptors with the highest Pearson correlation values between CITE-seq ADT data and abundance estimates produced by either the SPECK, ALRA, MAGIC or RNA transcript methods with number of cells specified to be 30,000 for the BMMC data, 37,000 for the Monocytes data, 20,000 for the mouse spleen data and 60,000 for the PBMC data. . . 5
S6 SPECK-based RRR and thresholded estimates versus SPECK-based RRR-only values and proportion of receptors with the highest Pearson correlation between CITE-seq ADT data and the estimated values for a subset of 30,000 cells for the BMMC data, 37,000 cells for the Monocytes data, 20,000 cells for the mouse spleen data and 60,000 cells for the PBMC data. 6
S7 Proportion of receptors with the highest Kendall's Tau correlation values between CITE-seq ADT data and abundance estimates produced by either the SPECK, ALRA, MAGIC or RNA transcript methods with number of cells specified to be 30,000 for the BMMC data, 37,000 for the Monocytes data, 20,000 for the mouse spleen data and 60,000 for the PBMC data. . . 7
S8 SPECK-based RRR and thresholded estimates versus SPECK-based RRR-only values and proportion of receptors with the highest Kendall's Tau correlation between CITE-seq ADT data and the estimated values for a subset of 30,000 cells for the BMMC data, 37,000 cells for the Monocytes data, 20,000 cells for the mouse spleen data and 60,000 cells for the PBMC data. 8
S9 Proportion of receptors with the maximum mean squared error (MSE) values between CITE-seq ADT data and abundance estimates produced by either the SPECK, ALRA, MAGIC or RNA transcript methods with number of cells specified to be 30,000 for the BMMC data, 37,000 for the Monocytes data, 20,000 for the mouse spleen data and 60,000 for the PBMC data. 9

S10 SPECK-based RRR and thresholded estimates versus SPECK-based RRR-only values and proportion of receptors with the maximum mean squared error (MSE) values between CITE-seq ADT data and the estimated values for a subset of 30,000 cells for the BMMC data, 37,000 cells for the Monocytes data, 20,000 cells for the mouse spleen data and 60,000 cells for the PBMC data. . . . 10
S11 Proportion of receptors with the maximum mean absolute error (MAE) values between CITE-seq ADT data and abundance estimates produced by either the SPECK, ALRA, MAGIC or RNA transcript methods with number of cells specified to be 30,000 for the BMMC data, 37,000 for the Monocytes data, 20,000 for the mouse spleen data and 60,000 for the PBMC data. . . . 11
S12 SPECK-based RRR and thresholded estimates versus SPECK-based RRR-only values and proportion of receptors with the maximum mean absolute error (MAE) values between CITE-seq ADT data and the estimated values for a subset of 30,000 cells for the BMMC data, 37,000 cells for the Monocytes data, 20,000 cells for the mouse spleen data and 60,000 cells for the PBMC data. . . . 12
S13 Individual Pearson correlations between CITE-seq ADT data and estimates generated by SPECK, ALRA, MAGIC and the RNA transcript method, averaged over a random subset of 60,000 cells for 215 receptors from the PBMC data. . . . 13
S14 Individual Pearson correlations between CITE-seq ADT data and estimates generated by SPECK, ALRA, MAGIC and the RNA transcript method, averaged over a random subset of 30,000 cells for 25 receptors from the BMMC data. . . . 14
S15 Individual Spearman rank correlations between CITE-seq ADT data and estimates generated by SPECK, ALRA, MAGIC and the RNA transcript method, averaged over a random subset of 37,000 cells for 238 receptors from the monocytes data. . . . 15
S16 Individual Pearson correlations between CITE-seq ADT data and estimates generated by SPECK, ALRA, MAGIC and the RNA transcript method, averaged over a random subset of 37,000 cells for 238 receptors from the monocytes data. . . . 16
S17 Individual Spearman rank correlations between CITE-seq ADT data and estimates generated by SPECK, ALRA, MAGIC and the RNA transcript method, averaged over a random subset of 20,000 cells for 102 receptors from the mouse spleen and lymph nodes data. . . . 17
S18 Individual Pearson correlations between CITE-seq ADT data and estimates generated by SPECK, ALRA, MAGIC and the RNA transcript method, averaged over a random subset of 20,000 cells for 102 receptors from the mouse spleen and lymph nodes data. . . . 18
S19 Low-dimensional projection of abundance profiles for CD14, CD19 and CD79b receptors as estimated by SPECK, ALRA, MAGIC and the RNA transcript method and corresponding CITE-seq ADT data for a subset of 10,000 cells from the monocytes data. . . . 19
S20 Smoothed scatter plot representation of the correspondence between CITE-seq ADT measurements for CD14, CD19 and CD79b and the receptor abundance profiles generated by SPECK, ALRA, MAGIC and the RNA transcript method for the monocytes data. . . . 19
S21 Low-dimensional projection of abundance profiles for CD14, CD19 and CD8A receptors as estimated by SPECK, ALRA, MAGIC and the RNA transcript method and corresponding CITE-seq ADT data for a subset of 10,000 cells from the mouse spleen and lymph nodes data. 20
S22 Smoothed scatter plot representation of the correspondence between CITE-seq ADT measurements for CD14, CD19 and CD8A and the receptor abundance profiles generated by SPECK, ALRA, MAGIC and the RNA transcript method for the mouse spleen and lymph nodes data. 21
S23 Clustered dendrogram representation of the BMMC-based Spearman rank correlation data. . 22

1 Supplemental Methods

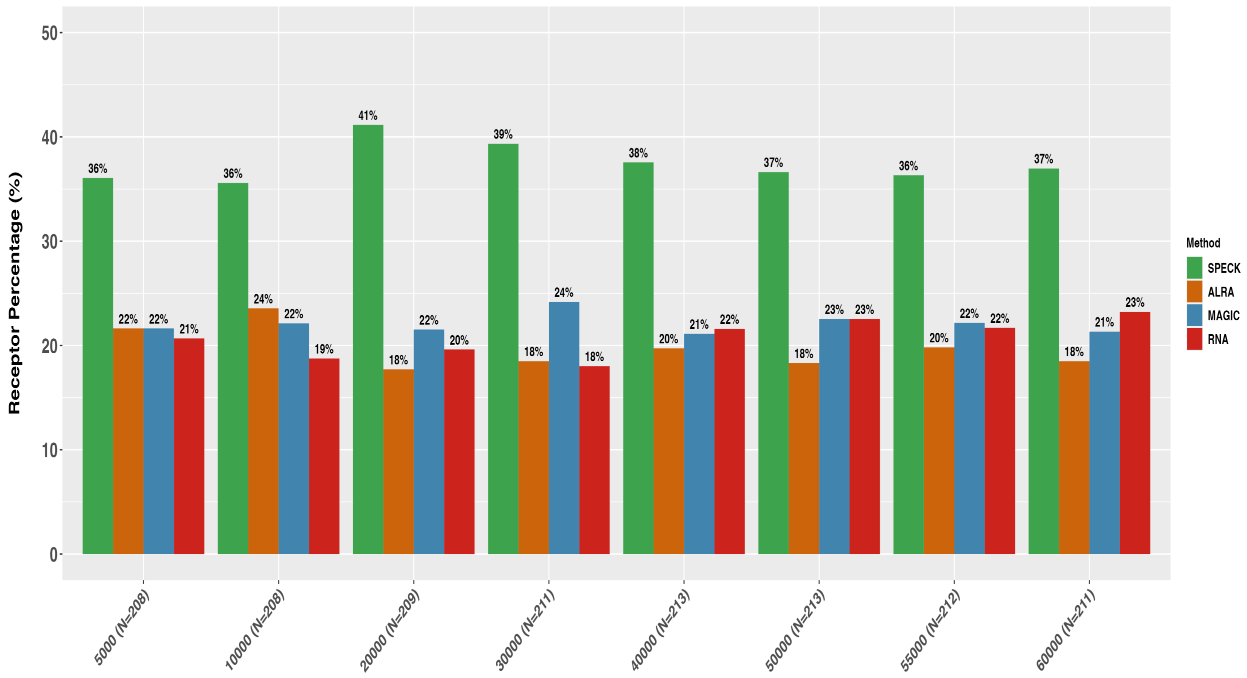

Figure S1: Proportion of receptors with the highest rank correlation values between CITE-seq ADT data and abundance estimates produced by either the SPECK, ALRA, MAGIC or RNA transcript methods with the number of cells ranging from 5,000 to 60,000 for the PBMC data.

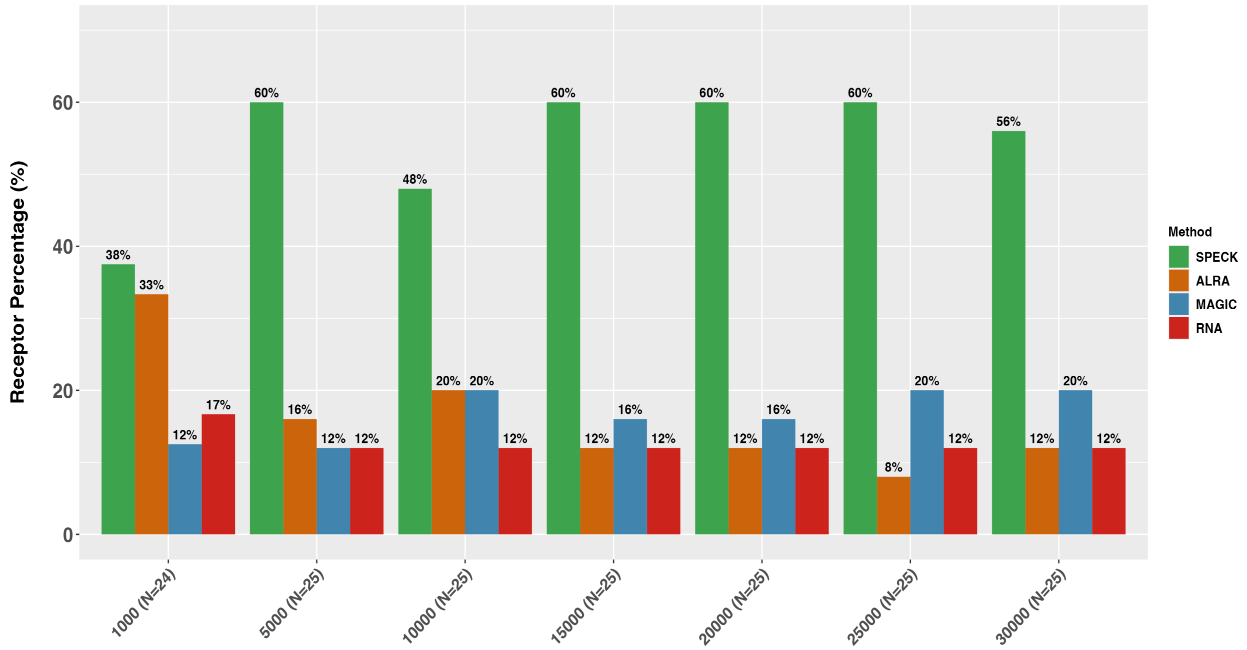

Figure S2: Proportion of receptors with the highest rank correlation values between CITE-seq ADT data and abundance estimates produced by either the SPECK, ALRA, MAGIC or RNA transcript methods with the number of cells ranging from 1,000 to 30,000 for the BMMC data.

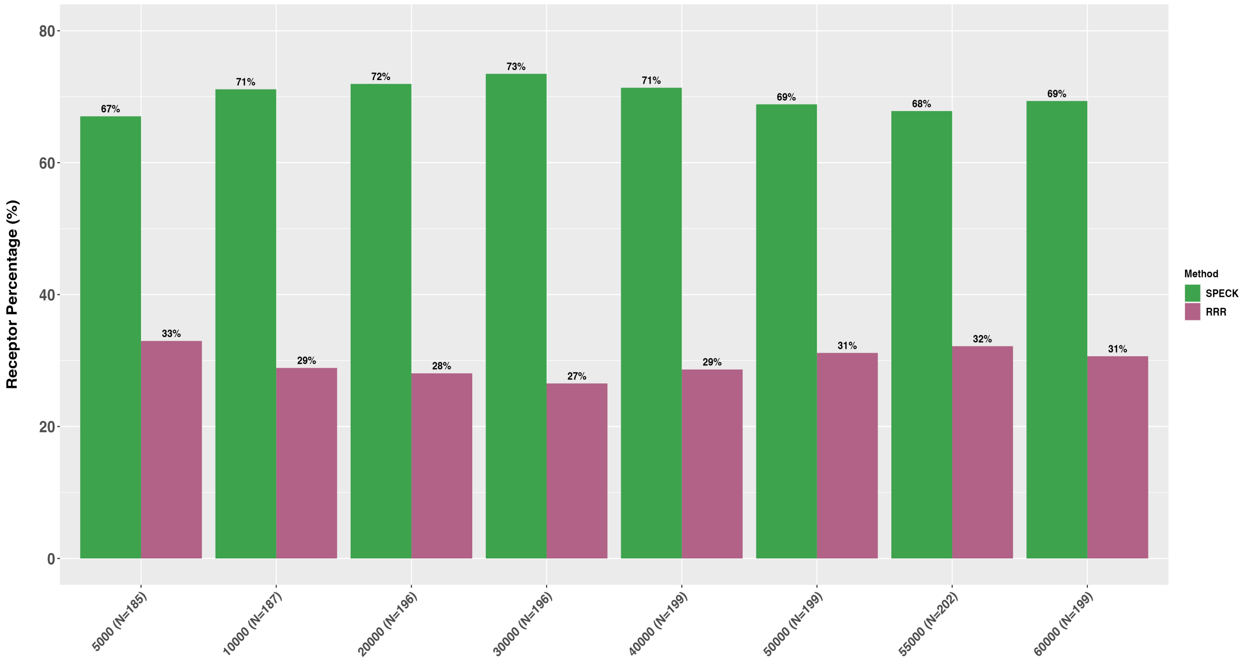

Figure S3: SPECK-based RRR and thresholded estimates versus SPECK-based RRR-only values and proportion of receptors with the highest Spearman rank correlation between CITE-seq ADT data and the estimated values for the PBMC data.

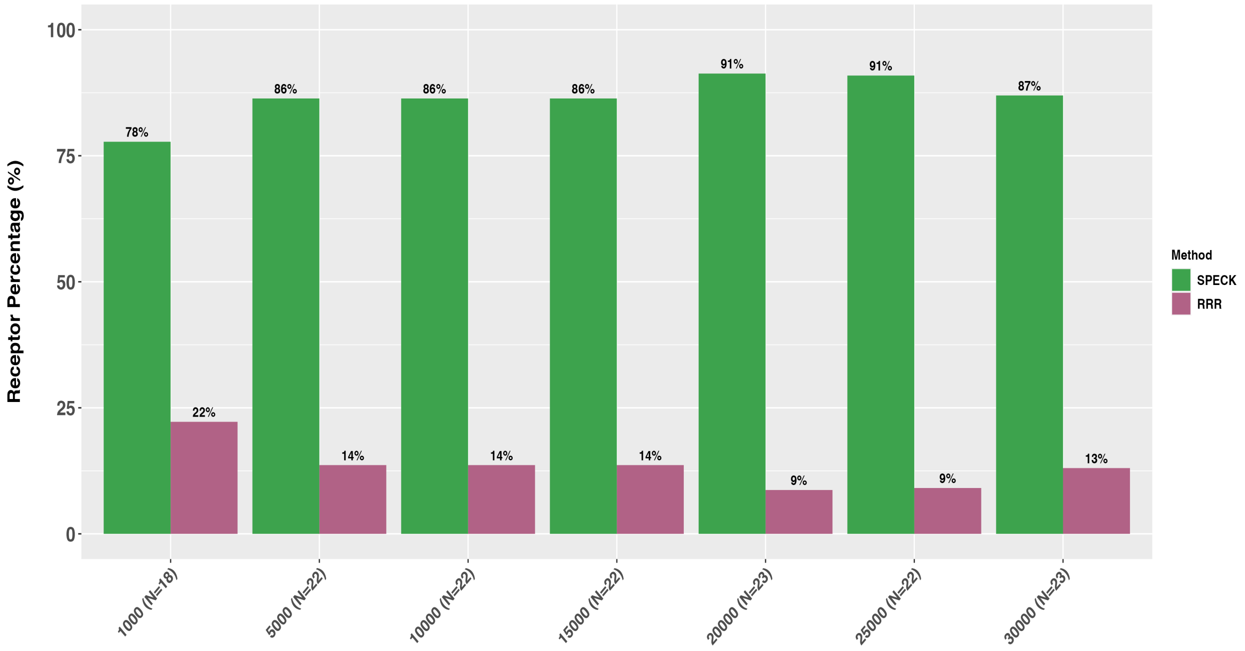

Figure S4: SPECK-based RRR and thresholded estimates versus SPECK-based RRR-only values and proportion of receptors with the highest Spearman rank correlation between CITE-seq ADT data and the estimated values for the BMMC data.

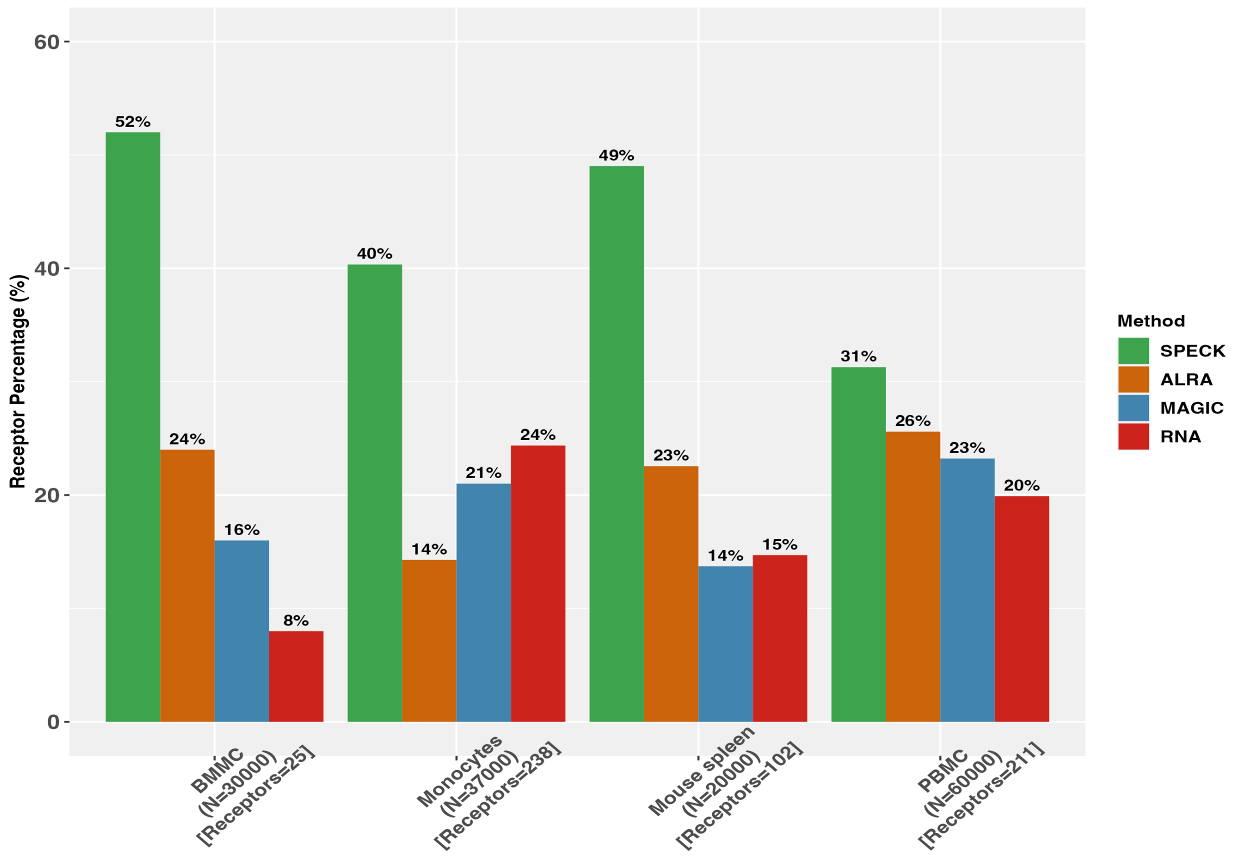

Figure S5: Proportion of receptors with the highest Pearson correlation values between CITE-seq ADT data and abundance estimates produced by either the SPECK, ALRA, MAGIC or RNA transcript methods with number of cells specified to be 30,000 for the BMMC data, 37,000 for the Monocytes data, 20,000 for the mouse spleen data and 60,000 for the PBMC data.

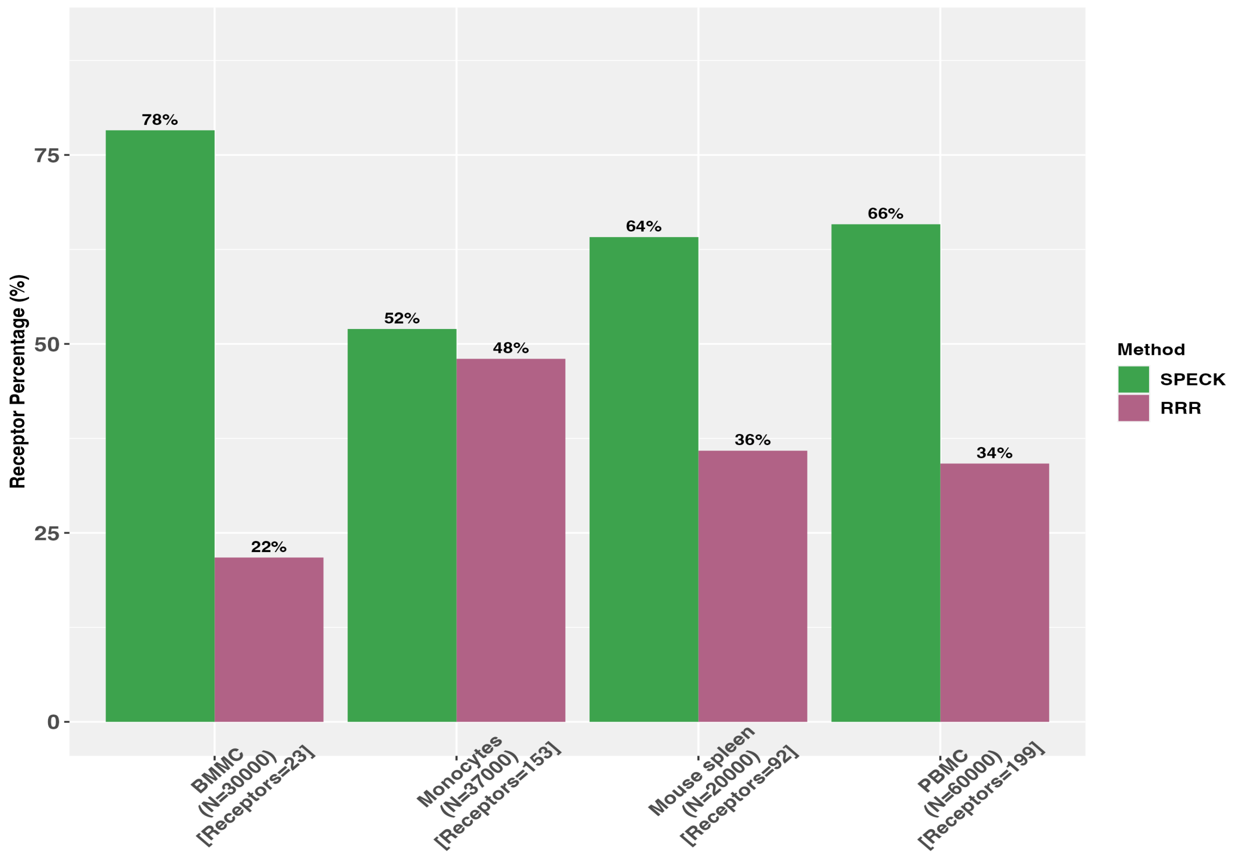

Figure S6: SPECK-based RRR and thresholded estimates versus SPECK-based RRR-only values and proportion of receptors with the highest Pearson correlation between CITE-seq ADT data and the estimated values for a subset of 30,000 cells for the BMMC data, 37,000 cells for the Monocytes data, 20,000 cells for the mouse spleen data and 60,000 cells for the PBMC data.

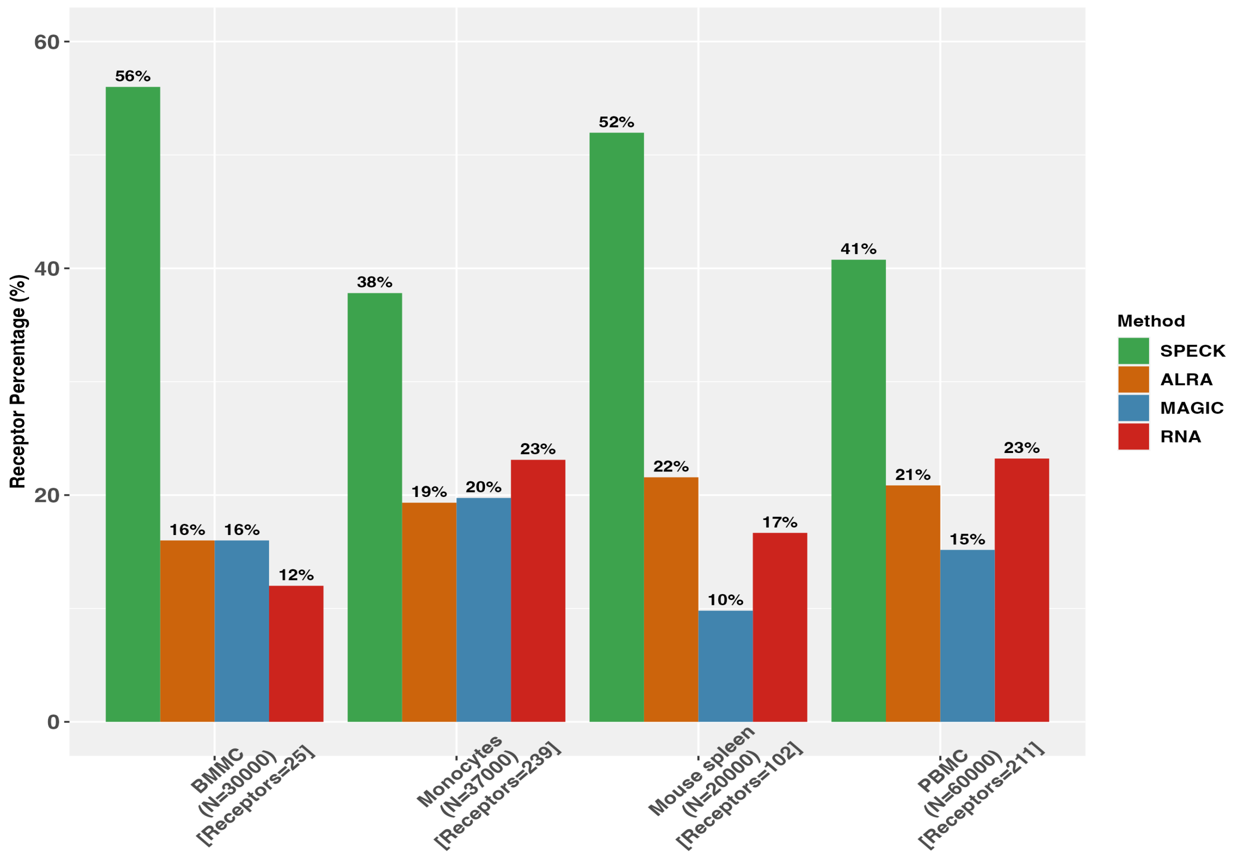

Figure S7: Proportion of receptors with the highest Kendall's Tau correlation values between CITE-seq ADT data and abundance estimates produced by either the SPECK, ALRA, MAGIC or RNA transcript methods with number of cells specified to be 30,000 for the BMMC data, 37,000 for the Monocytes data, 20,000 for the mouse spleen data and 60,000 for the PBMC data.

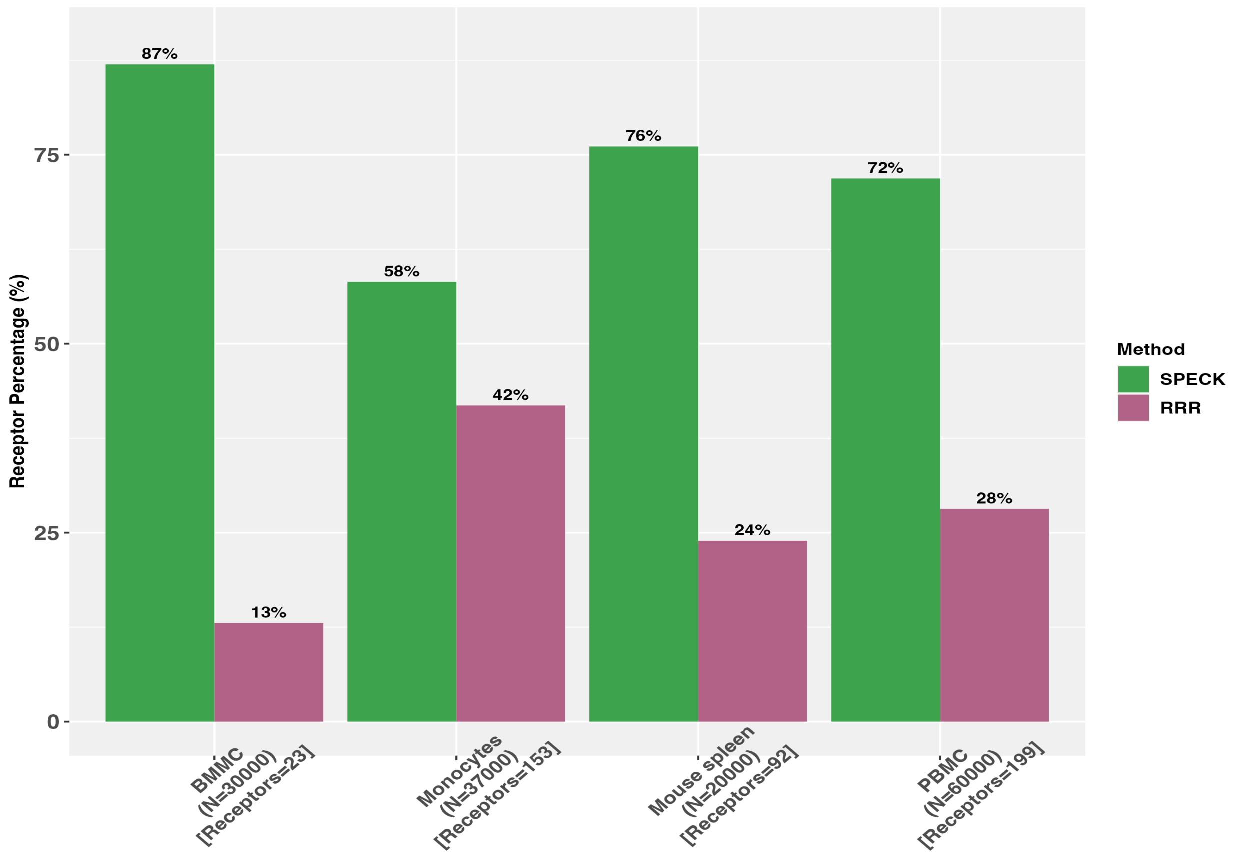

Figure S8: SPECK-based RRR and thresholded estimates versus SPECK-based RRR-only values and proportion of receptors with the highest Kendall's Tau correlation between CITE-seq ADT data and the estimated values for a subset of 30,000 cells for the BMMC data, 37,000 cells for the Monocytes data, 20,000 cells for the mouse spleen data and 60,000 cells for the PBMC data.

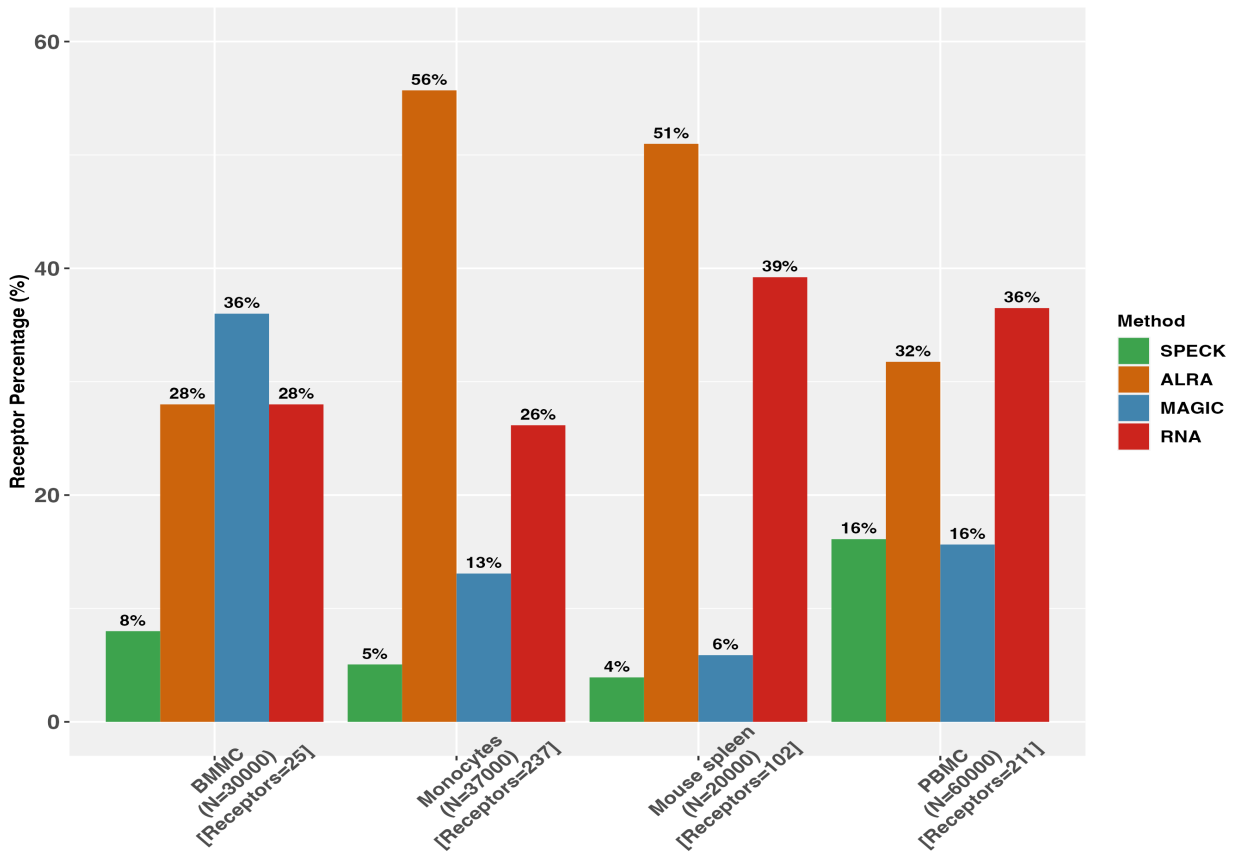

Figure S9: Proportion of receptors with the maximum mean squared error (MSE) values between CITE-seq ADT data and abundance estimates produced by either the SPECK, ALRA, MAGIC or RNA transcript methods with number of cells specified to be 30,000 for the BMMC data, 37,000 for the Monocytes data, 20,000 for the mouse spleen data and 60,000 for the PBMC data.

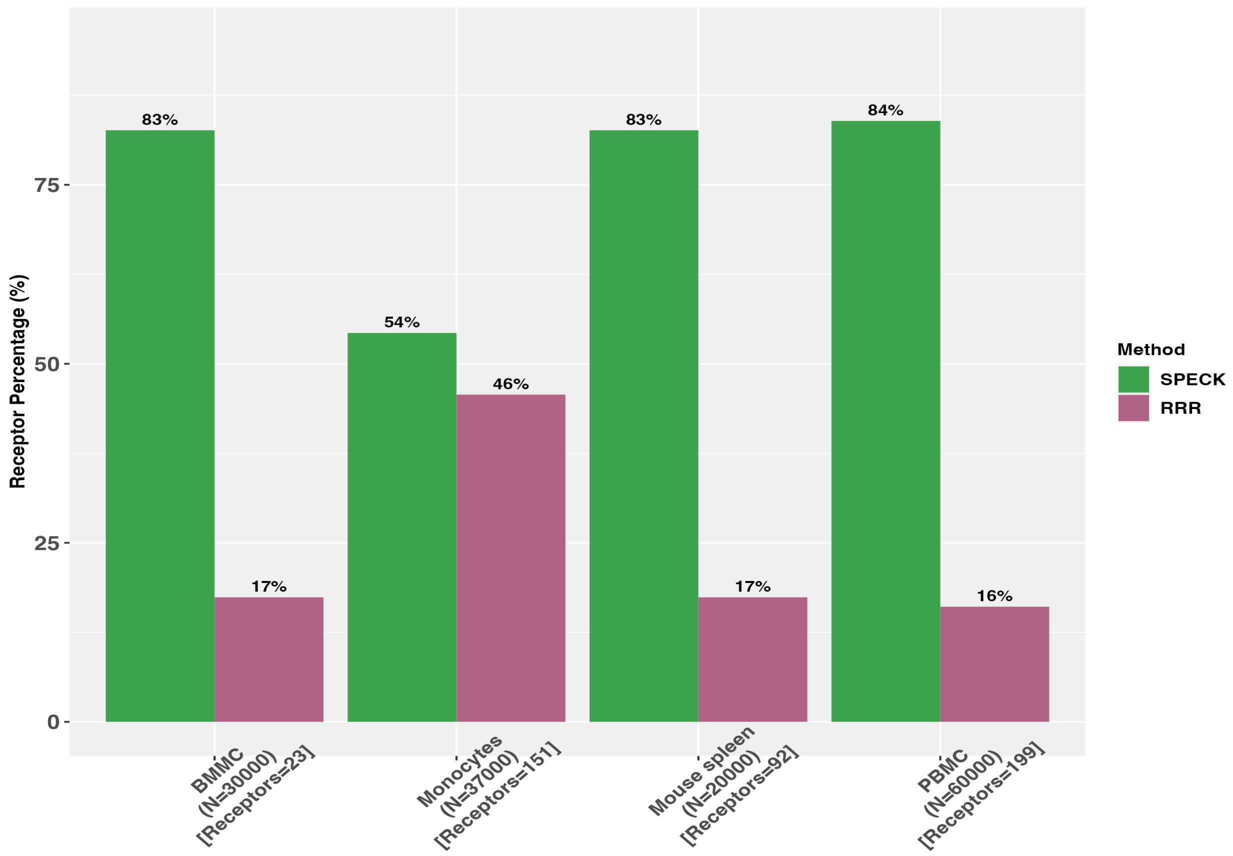

Figure S10: SPECK-based RRR and thresholded estimates versus SPECK-based RRR-only values and proportion of receptors with the maximum mean squared error (MSE) values between CITE-seq ADT data and the estimated values for a subset of 30,000 cells for the BMMC data, 37,000 cells for the Monocytes data, 20,000 cells for the mouse spleen data and 60,000 cells for the PBMC data.

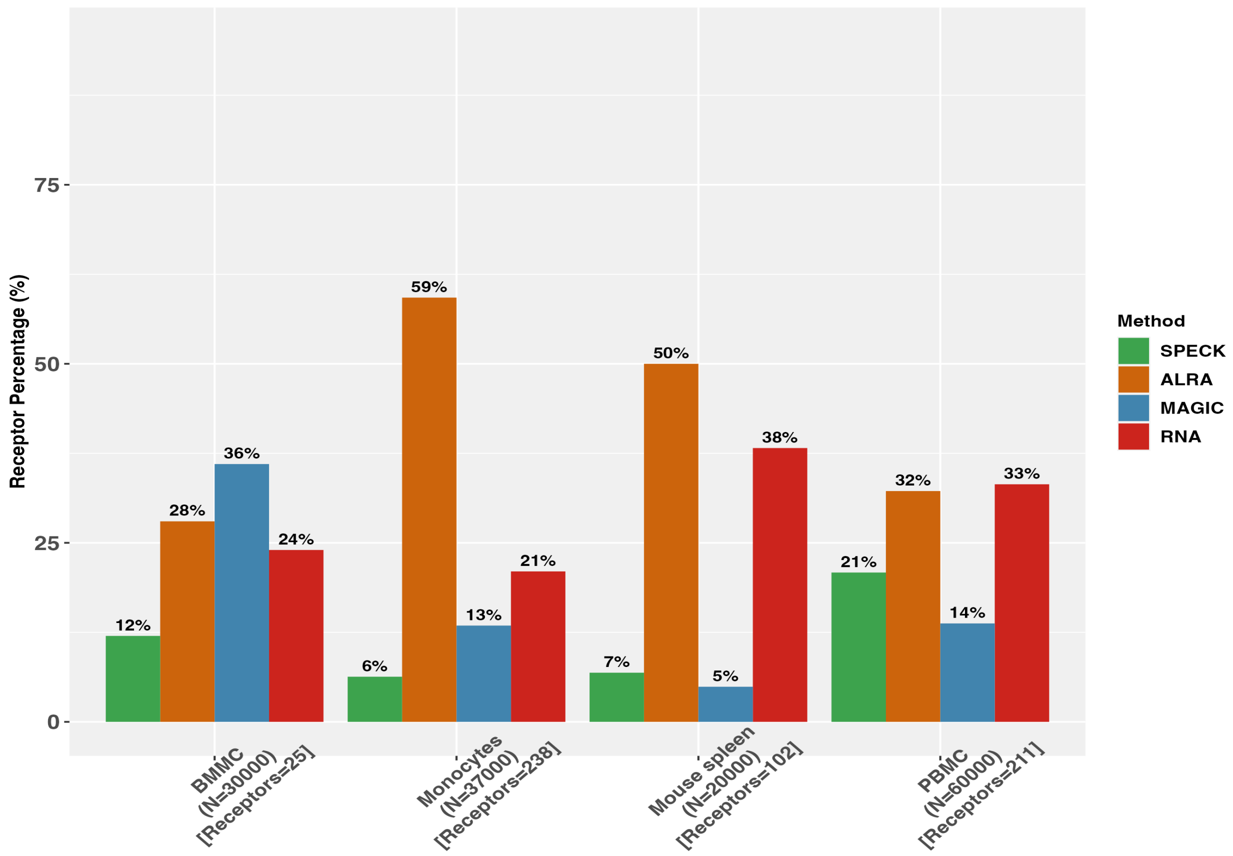

Figure S11: Proportion of receptors with the maximum mean absolute error (MAE) values between CITE-seq ADT data and abundance estimates produced by either the SPECK, ALRA, MAGIC or RNA transcript methods with number of cells specified to be 30,000 for the BMMC data, 37,000 for the Monocytes data, 20,000 for the mouse spleen data and 60,000 for the PBMC data.

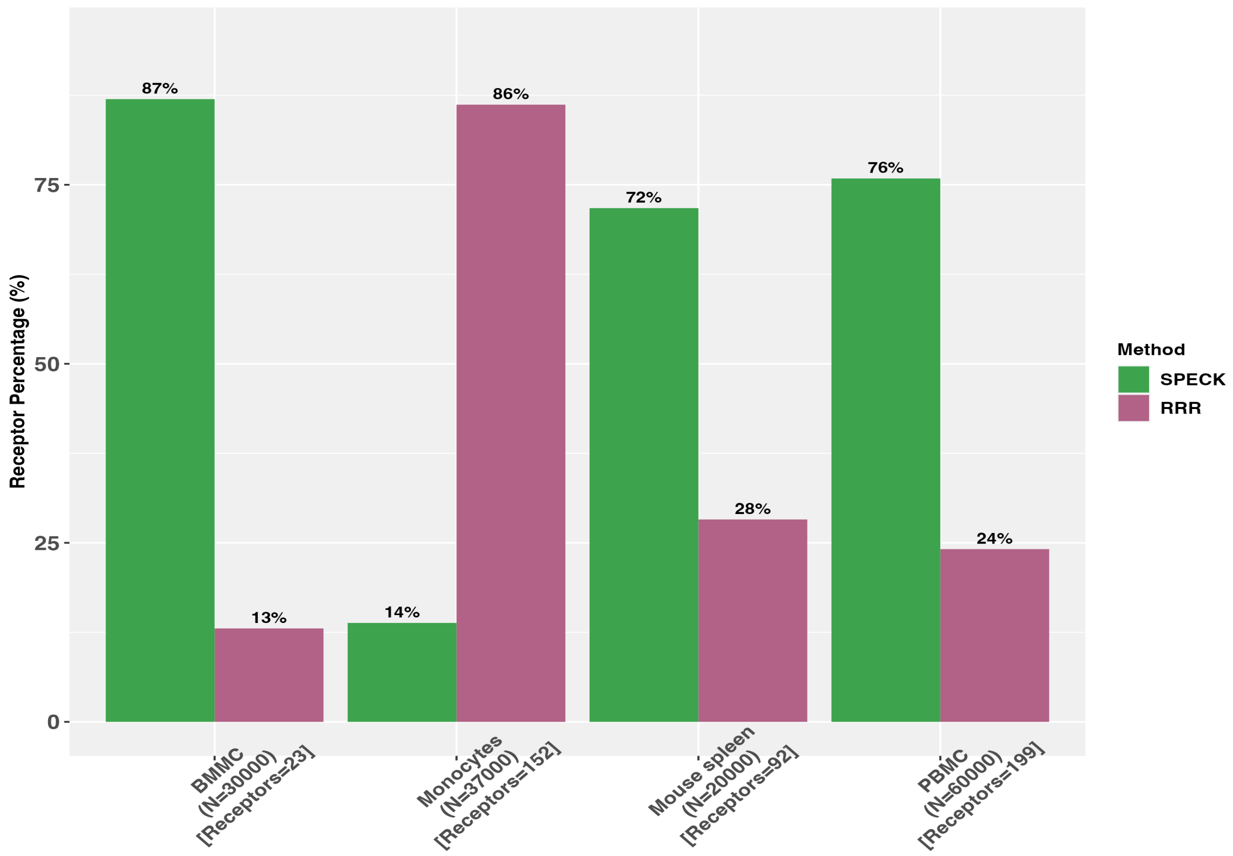

Figure S12: SPECK-based RRR and thresholded estimates versus SPECK-based RRR-only values and proportion of receptors with the maximum mean absolute error (MAE) values between CITE-seq ADT data and the estimated values for a subset of 30,000 cells for the BMMC data, 37,000 cells for the Monocytes data, 20,000 cells for the mouse spleen data and 60,000 cells for the PBMC data.

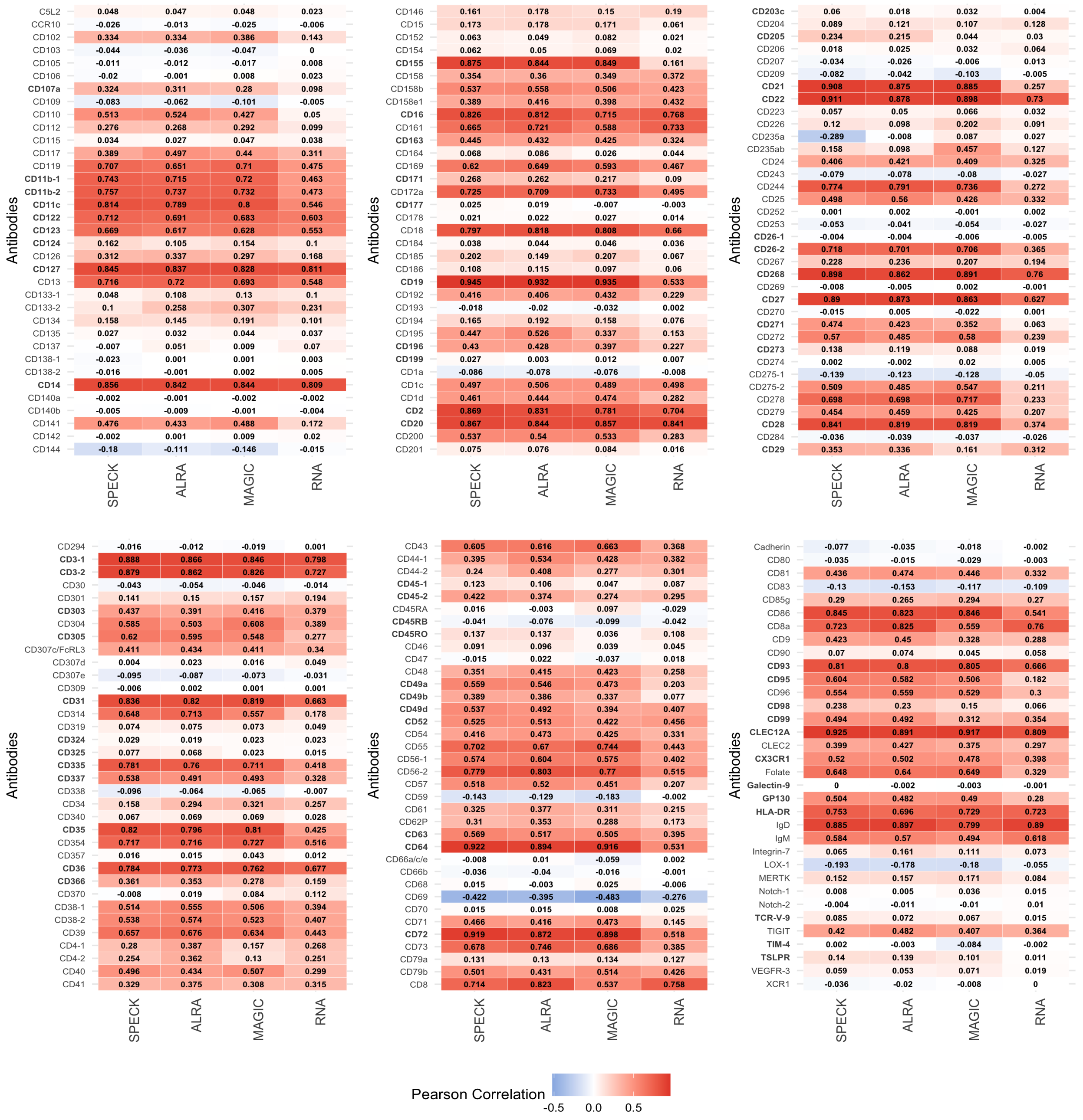

Figure S13: Individual Pearson correlations between CITE-seq ADT data and estimates generated by SPECK, ALRA, MAGIC and the RNA transcript method, averaged over a random subset of 60,000 cells for 215 receptors from the PBMC data.

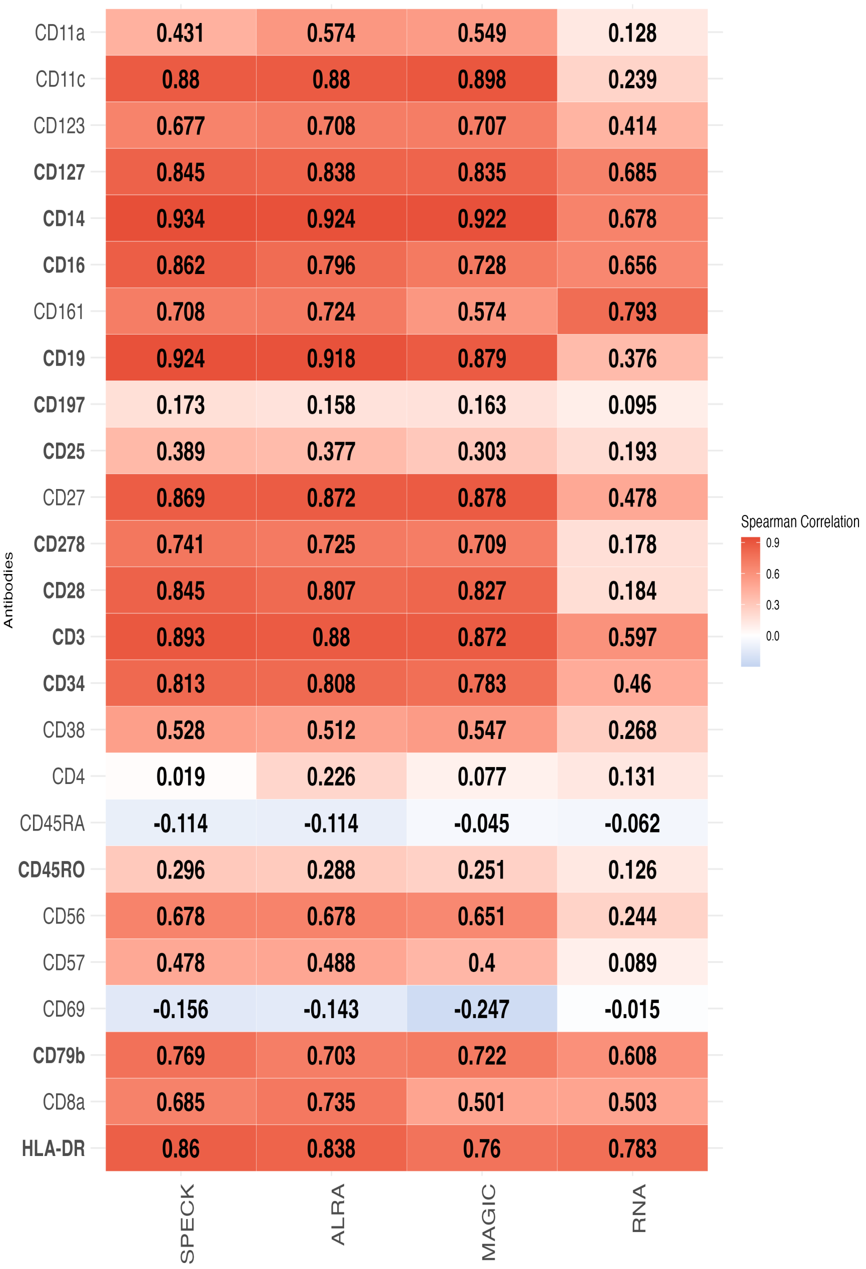

Figure S14: Individual Pearson correlations between CITE-seq ADT data and estimates generated by SPECK, ALRA, MAGIC and the RNA transcript method, averaged over a random subset of 30,000 cells for 25 receptors from the BMMC data.

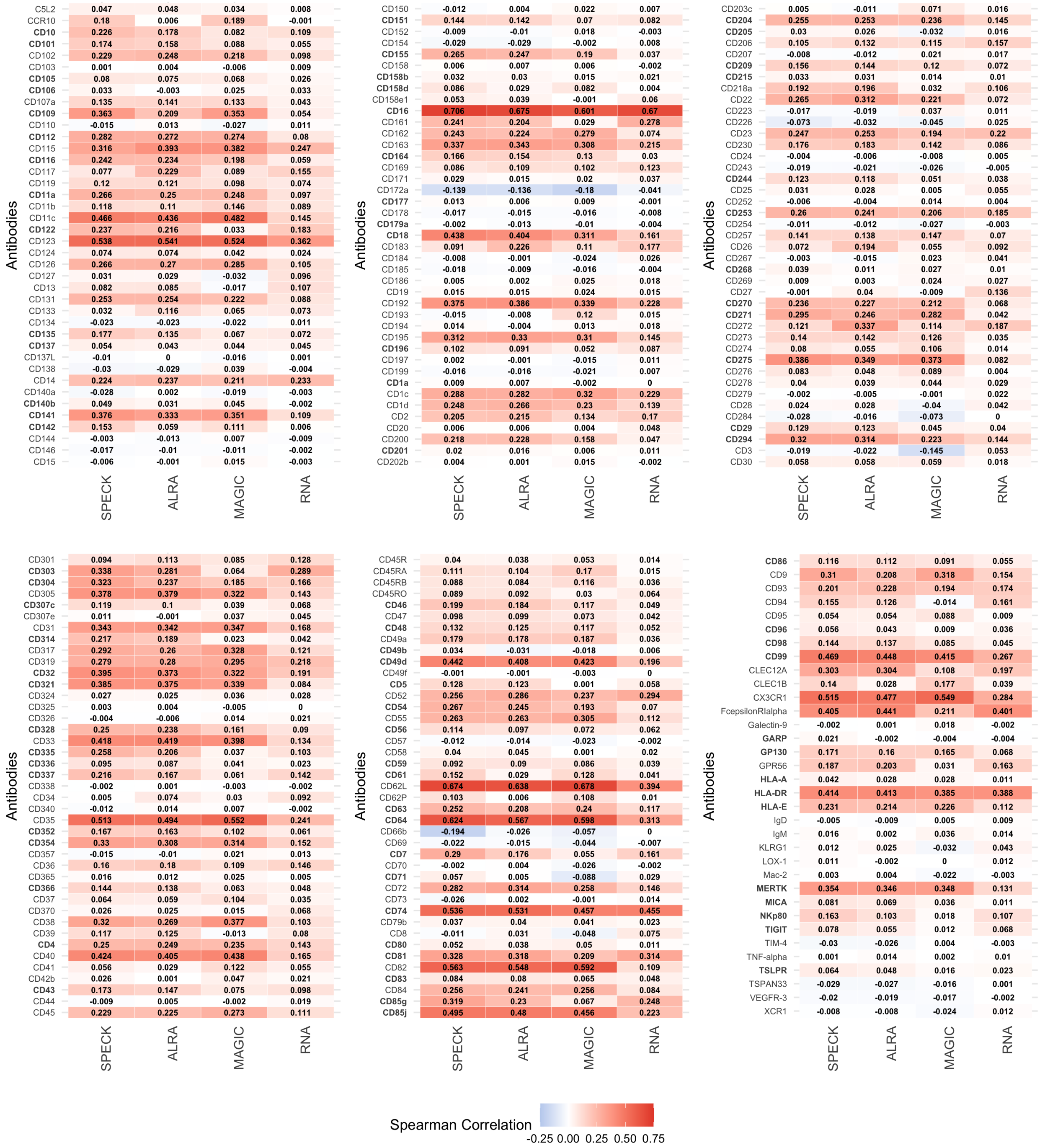

Figure S15: Individual Spearman rank correlations between CITE-seq ADT data and estimates generated by SPECK, ALRA, MAGIC and the RNA transcript method, averaged over a random subset of 37,000 cells for 238 receptors from the monocytes data.

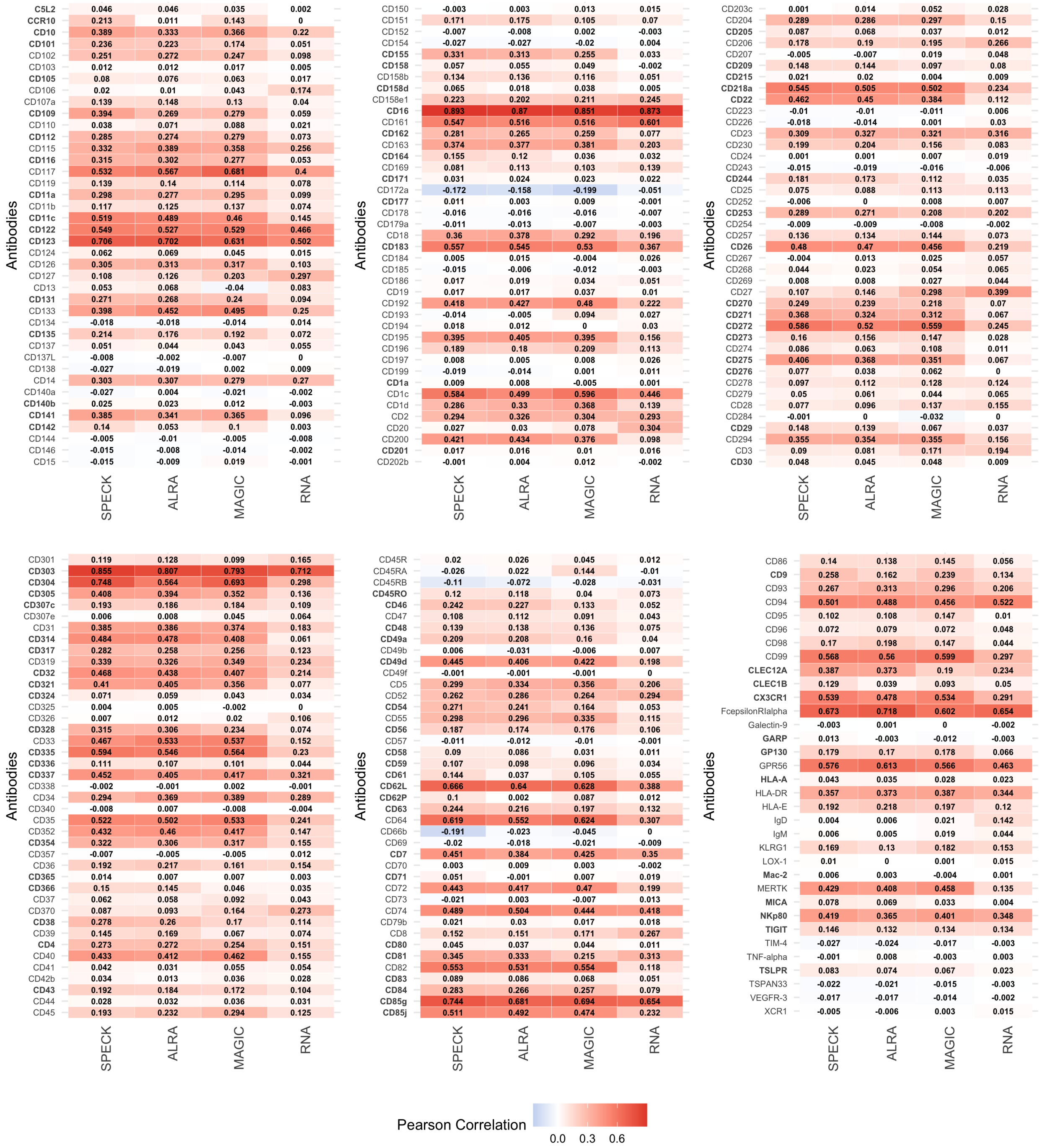

Figure S16: Individual Pearson correlations between CITE-seq ADT data and estimates generated by SPECK, ALRA, MAGIC and the RNA transcript method, averaged over a random subset of 37,000 cells for 238 receptors from the monocytes data.

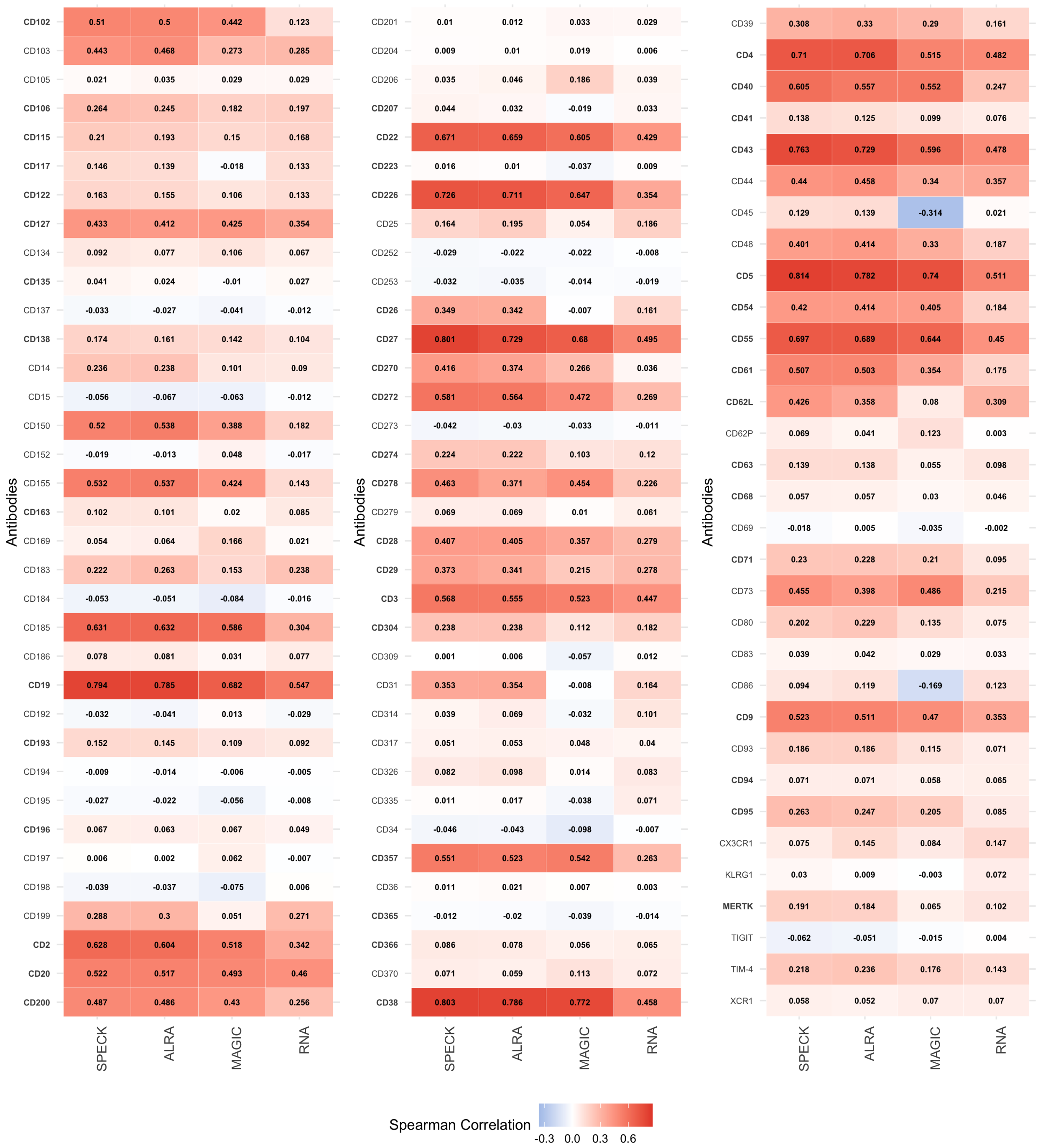

Figure S17: Individual Spearman rank correlations between CITE-seq ADT data and estimates generated by SPECK, ALRA, MAGIC and the RNA transcript method, averaged over a random subset of 20,000 cells for 102 receptors from the mouse spleen and lymph nodes data.

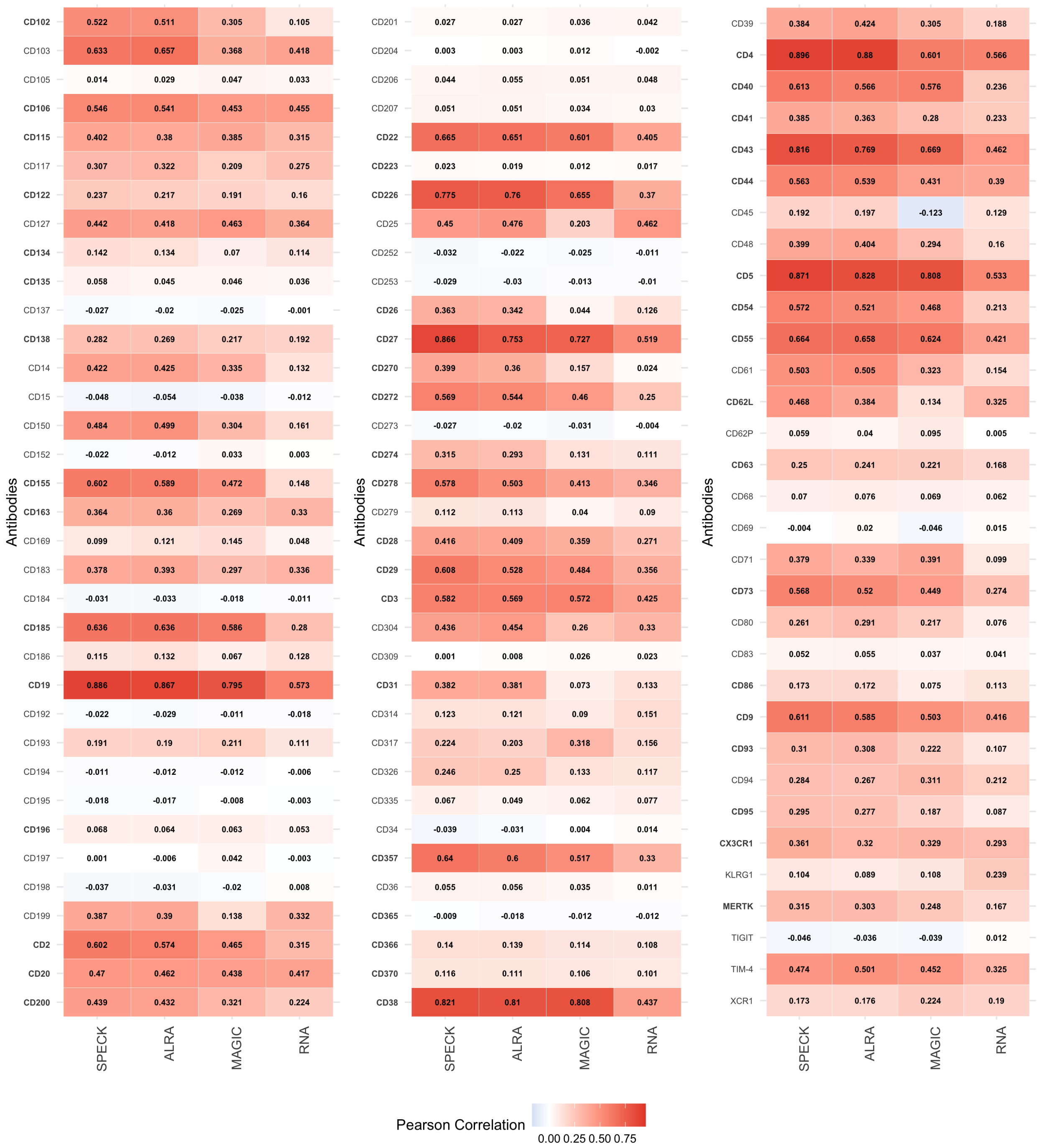

Figure S18: Individual Pearson correlations between CITE-seq ADT data and estimates generated by SPECK, ALRA, MAGIC and the RNA transcript method, averaged over a random subset of 20,000 cells for 102 receptors from the mouse spleen and lymph nodes data.

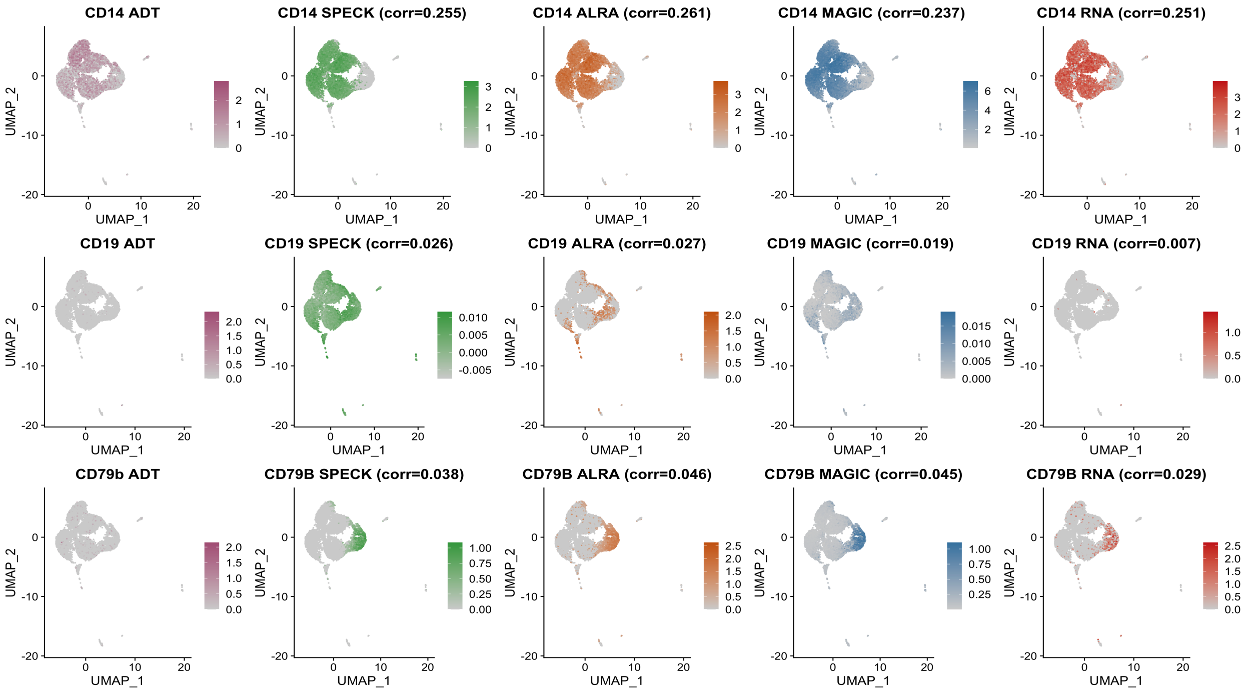

Figure S19: Low-dimensional projection of abundance profiles for CD14, CD19 and CD79b receptors as estimated by SPECK, ALRA, MAGIC and the RNA transcript method and corresponding CITE-seq ADT data for a subset of 10,000 cells from the monocytes data.

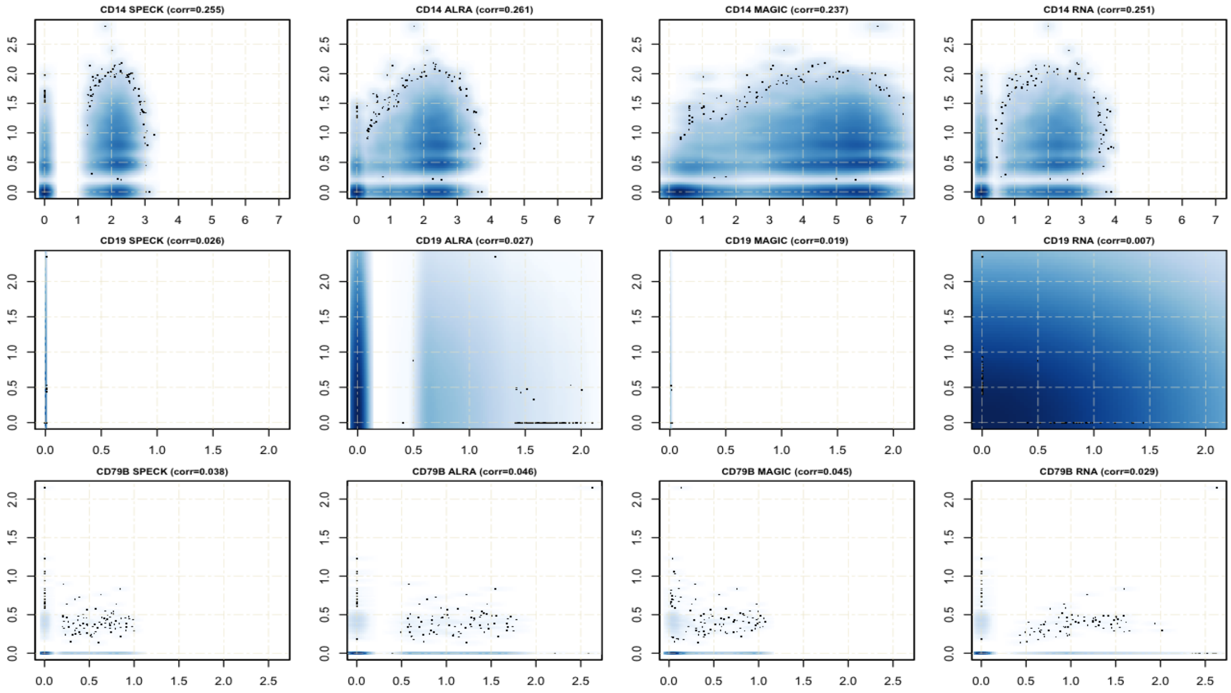

Figure S20: Smoothed scatter plot representation of the correspondence between CITE-seq ADT measurements for CD14, CD19 and CD79b and the receptor abundance profiles generated by SPECK, ALRA, MAGIC and the RNA transcript method for the monocytes data.

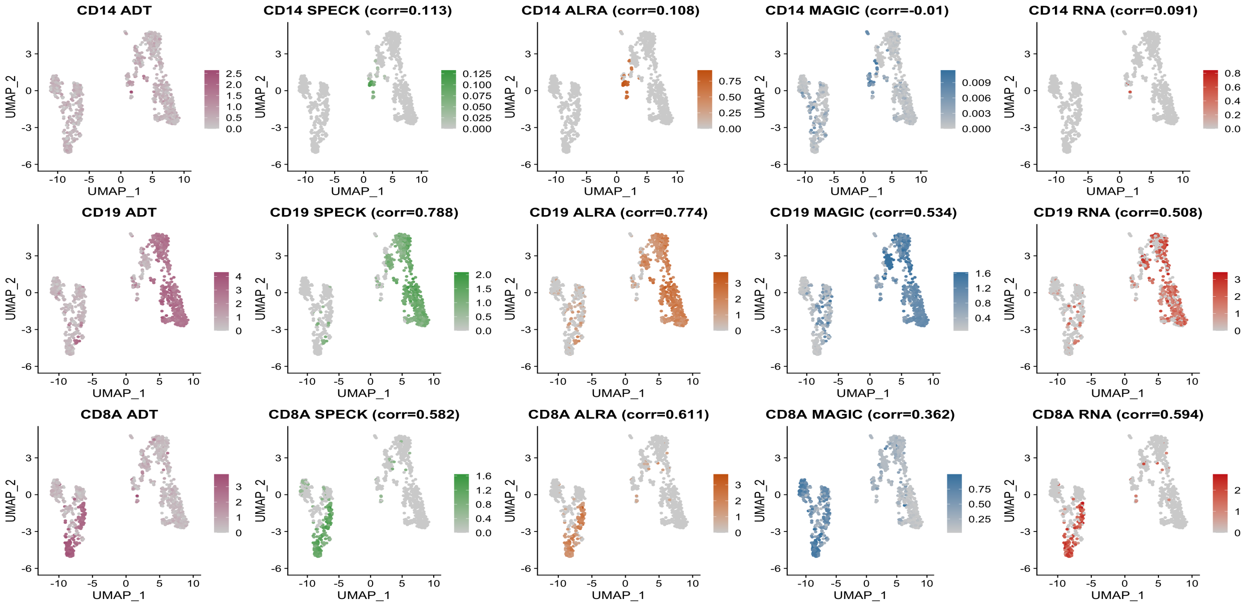

Figure S21: Low-dimensional projection of abundance profiles for CD14, CD19 and CD8A receptors as estimated by SPECK, ALRA, MAGIC and the RNA transcript method and corresponding CITE-seq ADT data for a subset of 10,000 cells from the mouse spleen and lymph nodes data.

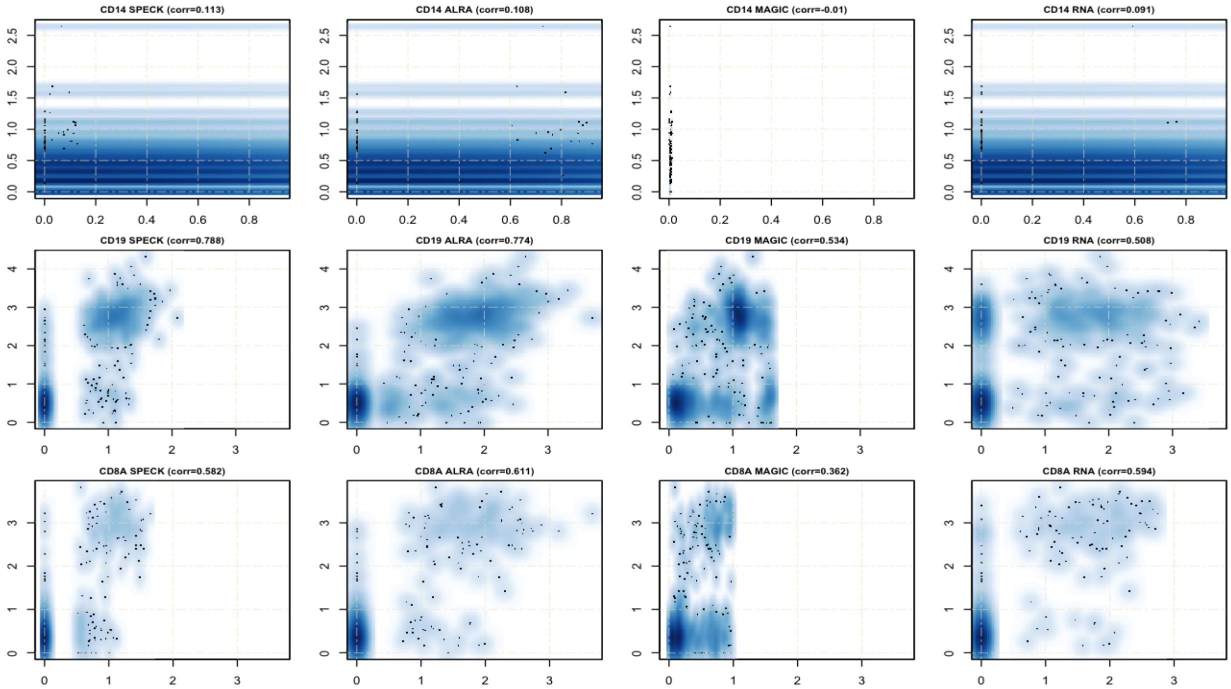

Figure S22: Smoothed scatter plot representation of the correspondence between CITE-seq ADT measurements for CD14, CD19 and CD8A and the receptor abundance profiles generated by SPECK, ALRA, MAGIC and the RNA transcript method for the mouse spleen and lymph nodes data.

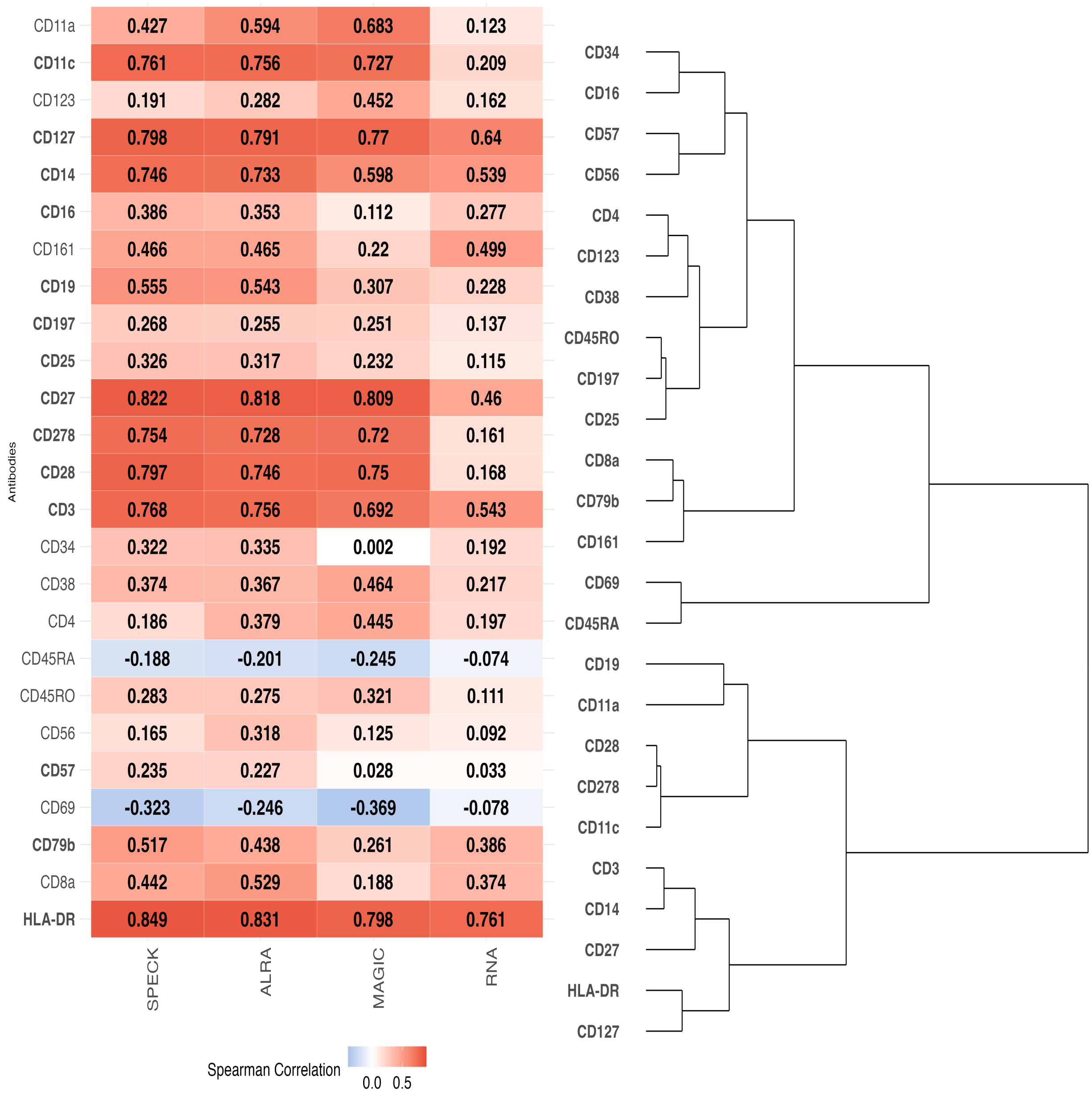

Figure S23: Clustered dendrogram representation of the BMMC-based Spearman rank correlation data.
